# Direct Determination of a Non-Cooperative LEO Satellite’s Orbital State from Ground-Station Signals: A Cramér–Rao Analysis

**DOI:** 10.3390/s26196306

**Published:** 2026-10-06

**Authors:** Anthony J. Weiss

**Affiliations:** School of Electrical Engineering, Tel Aviv University, Tel Aviv 6997801, Israel; tony@tauex.tau.ac.il

**Keywords:** direct position determination, orbital-state estimation, Cramér–Rao bound, LEO satellites, passive geolocation, non-cooperative emitters, two-body dynamics, state-transition matrix, space situational awareness

## Abstract

This paper considers passive determination of the orbital state of a non-cooperative low-Earth-orbit satellite from radio signals received at several ground stations. Reversing the roles of direct geolocation in which a satellite with known ephemeris locates a ground emitter, the satellite’s six-dimensional epoch state is estimated in a single step from the received data. Because the trajectory is a deterministic function of this state, every candidate is a valid orbit. The Cramér–Rao bound is derived; it factors into signal, geometry and orbital-dynamics terms, and a closed-form Keplerian state-transition block is obtained by implicit differentiation of the universal Kepler equation. The delay model includes the light-time correction, which leaves the bound essentially unchanged but removes a bias of more than a hundred meters. The unknown emission epoch is treated as a nuisance; it roughly doubles the three-station bound, whereas four stations recover the known-epoch accuracy. Simulations validate the dynamics implementation and demonstrate post-acquisition efficiency under a Gaussian delay/Doppler surrogate of the known-waveform model. Under an independent-cell model of acquisition failures, an ideal exhaustive search predicts a threshold about 6 dB below that of a two-step baseline with hard per-window peak decisions. The paper also quantifies how accuracy scales with arc length, station count and station layout.

## 1. Introduction

The rapid growth of low-Earth-orbit (LEO) constellations has made independent non-cooperative determination of satellite orbits from their radio emissions a problem of practical interest for space situational awareness, interference geolocation, and spectrum monitoring. High-precision orbit determination (POD) can achieve centimeter-level accuracy [1,2,3] but relies on cooperative signals (e.g., DORIS, GNSS, and satellite laser ranging) and a two-step processing chain: range/range-rate observables are extracted per station and then an orbit is fitted to them. Two-step processing can lose information through intermediate observable extraction, particularly when the extracted observables become unreliable at low SNR, where they cannot be reliably extracted [4,5]. The same two-step structure underlies classical TDOA/FDOA emitter geolocation in which delay and Doppler differences are first measured by cross-correlation or cross-ambiguity processing [6,7] and the source is then located algebraically or by iterative fitting [8,9] and the recent passive RF tracking of satellites by synchronized ground-receiver networks in which TDOA and FDOA measurements feed an initial orbit determination [10,11].

Direct position determination (DPD)—also called direct geolocation—removes the intermediate stage by mapping raw multi-receiver I/Q data straight to position through a single cost function [4,5,12]; related single-step formulations include TDOA-based direct positioning with its maximum-likelihood estimator and CRB [13], the joint detection and localization of intermittent emitters [14], and direct position estimation in GNSS receivers [15]. DPD is known to outperform two-step methods at low SNR and short data captures [4,12,13]. Recent work has applied DPD from LEO to geolocate terrestrial emitters [16], and Doppler and Doppler-rate measurements from a single LEO satellite have been used to geolocate ground emitters [17]. In all of this literature, however, the satellite trajectory is assumed known from ephemeris; the satellite is the sensor, and the ground emitter is the unknown.

A separate and rapidly growing body of literature treats non-cooperative LEO satellites as signals of opportunity for positioning, navigation and timing, and there the satellite orbit is a nuisance that must be determined or corrected [18,19]. Doppler-based opportunistic positioning from LEO downlinks, including differential schemes and the analysis of their baseline dependence, is developed in [20,21,22,23,24,25]; orbit determination and ephemeris tracking of non-cooperative satellites from Doppler observables extracted by a receiver at a known position, including the simultaneous tracking-and-navigation framework and its machine learning variants, in [26,27,28,29,30,31]; and the modeling and compensation of the timing and spatial ephemeris errors of such satellites in [32,33]. All of these are two-step: per-station delay and/or Doppler observables are extracted from the signal and an orbit or a receiver state is then fitted to them, with the extraction usually relying on knowledge of the signal structure (a beacon, a pilot, or a modulation format). The present work differs in three respects, which are stated here so that the novelty is not misread: (i) the estimand is the six-dimensional orbital state of the emitter with the receivers at known positions—the inverse of the receiver-localization problem—so that the satellite is treated exactly as a moving emitter in direct geolocation; (ii) the estimation is single-step, from the received samples, with the orbital dynamics built into the parameterization rather than imposed on extracted observables; and (iii) the Cramér–Rao bound is derived in a form that separates signal, geometry and dynamics, with the dynamics entering through a closed-form Keplerian state-transition block. The first two are what make the low-SNR behavior different from that of the two-step chain; the third is what makes the accuracy predictable. Direct processing of raw data (the DPD principle) and the inversion of roles are the setting rather than the novelty in themselves; the principal contributions are items (ii) and (iii): the incorporation of the orbital dynamics into the single-step parameterization, and the resulting factorized orbital-state CRB with its closed-form dynamics term.

This paper reverses the roles of the direct-geolocation configuration of [4,5,16,17]: instead of a satellite with known ephemeris locating an unknown ground emitter, ground stations at known positions determine the unknown six-dimensional orbital state of a non-cooperative satellite emitter. The contributions are:

1. A single-step estimator that determines the orbital state directly from the received signals in which the orbital-dynamics manifold is intrinsic to the parameterization. Every candidate is a valid orbit, so the entire trajectory, rather than a sequence of per-epoch positions, is searched through six orbital parameters (plus nuisances where present) independent of the arc length and the number of samples with no constraint penalty (Section 3);
2. A closed-form Cramér–Rao bound for the orbital state, with the light-time correction included in the delay model, that factors into signal, geometry, and dynamics terms, the dynamics entering as the two-body state-transition matrix (STM), with the Keplerian STM position block derived in closed form by implicit differentiation of the universal Kepler equation (Section 4 and Section 5);
3. Numerical validation of the derivation and known-waveform estimator efficiency, an observability analysis with a nondimensionalized state over several station layouts and passes, and a model-based comparison of the acquisition (wrong-peak) thresholds of the direct estimator and a two-step baseline that commits to hard per-window peak decisions (Section 7).

The approach is distinct from angle-only non-cooperative orbit determination (which uses optical bearings and two-step filtering) and POD (cooperative two-step); it is the direct RF single-step counterpart aimed at the weak and uncooperative regime.

## 2. Signal and Geometry Model

Let the unknown orbital state at epoch $t_{0}$ be

(1) $$x=\left[\begin{array}{c} r_{0}\\ v_{0} \end{array}\right]\in R^{6},$$

the satellite position $r_{0}$ and velocity $v_{0}$ at the epoch $t_{0}$, expressed in an Earth-centered inertial (ECI) frame. The ECI realization used throughout is the simplest one that is adequate for a single pass of a few minutes: it accounts for Earth’s rotation about its polar axis at the sidereal rate $\omega_{⊕}$ and neglects precession, nutation and polar motion, whose effects over the observation intervals considered are far below the accuracies of interest. For processing real data with published ephemerides, the full transformation between the terrestrial (ITRF) and celestial (GCRF) frames with the IAU precession–nutation and polar-motion models would be used [2,3]. Both the satellite and the ground stations are expressed in this frame; a station fixed in Earth-centered Earth-fixed (ECEF) coordinates therefore has a time-varying ECI position $q_{m}\left(t\right)=R_{⊕}\left(t\right)\;q_{m}^{\mathrm{ECEF}}$, where $R_{⊕}\left(t\right)$ is the rotation about the polar axis by the angle $\omega_{⊕}\left(t-t_{0}\right)$, and a corresponding ECI velocity ${\dot{q}}_{m}\left(t\right)=\omega_{⊕}\times q_{m}\left(t\right)$ of a few hundred m/s, which enters the Doppler through the relative range rate ${\dot{\tau }}_{m}=\frac{1}{c}{\hat{u}}_{m}^{⊤}\left(v_{s}-{\dot{q}}_{m}\right)$, with $v_{s}\left(t\right)$ the satellite velocity and ${\hat{u}}_{m}\left(t\right)$ the unit line-of-sight vector defined below, and is retained throughout. Under the adopted dynamical model the satellite position $p_{s}\left(t\right)$ is a deterministic function of the epoch state (for two-body motion in closed form; for perturbed motion by numerical integration of the equations of motion, which is deterministic once the force model is fixed). Stochastic accelerations are not modeled here; when process noise is modeled, the appropriate bound is the posterior (Bayesian) CRB of nonlinear filtering [34,35],

(2) $$p_{s}\left(t\right)=\phi \left(x,t\right),\;\phi \left(x,t_{0}\right)=r_{0}.$$

Ground station m=1,…,M receives the baseband signal

(3) $$y_{m}\left(t\right)=b_{m}\;s\;\left(t-\tau_{m}\left(t\right)\right)\;e^{-j2\pi f_{c}\tau_{m}\left(t\right)}+w_{m}\left(t\right),\;w_{m}\;∼\;\mathrm{CN}\left(0,N_{0}\right),$$

where s(·) is the (initially known) complex envelope, $f_{c}$ the carrier, $b_{m}$ a (constant) complex gain, and *c* the speed of light. For a non-cooperative emitter the emission epoch is not known to the receivers, so the envelope in (3) is more generally $s\left(t-\tau_{m}\left(t\right)-\delta t_{\mathrm{tx}}\right)$, with $\delta t_{\mathrm{tx}}$ an unknown time offset common to all stations. Like the absolute carrier phase absorbed in $b_{m}$, $\delta t_{\mathrm{tx}}$ is not separable from the station clock offsets (Section 4); with the stations synchronized to a common reference it is a single common nuisance, and the delay observable then carries only time-difference information across stations while the Doppler and Doppler rate are unaffected. For clarity of exposition $\delta t_{\mathrm{tx}}$ is suppressed in (3)–(9) and restored as a nuisance in Section 4. In this constant-gain model the geometric dependence is represented through the time-varying propagation delay, defined implicitly by the light-time equation

(4) $$\;\tau_{m}\left(t\right)=\frac{1}{c}∥\phi \left(x,\;t-\tau_{m}\left(t\right)\right)-q_{m}\left(t\right)∥\;,$$

i.e., the signal received at *t* was emitted at the retarded epoch $t_{e}=t-\tau_{m}\left(t\right)$ from the satellite position at that epoch. The propagation time $\tau_{m}$, termed the one-way light time in orbit determination [2,36] (propagation time has also been modeled explicitly in LEO Doppler positioning [25]) (as opposed to the two-way light time of active ranging), is about 2–3.5 ms at the ranges of the observed arc, during which the satellite moves 14–25 m, and Section 7 shows that evaluating the satellite position at the reception time instead would leave the bound essentially unchanged but bias the estimate by more than a hundred meters. For given x and *t*, (4) is an implicit equation for the delay $\tau_{m}\left(t\right)$; it is solved by fixed-point iteration, starting from the simultaneous-time value $\tau_{m}^{\left(0\right)}=∥\phi \left(x,t\right)-q_{m}\left(t\right)∥/c$ and iterating $\tau_{m}^{\left(k+1\right)}=∥\phi \left(x,t-\tau_{m}^{\left(k\right)}\right)-q_{m}\left(t\right)∥/c$. Because the iteration contracts by the factor $|{\hat{u}}_{m}^{⊤}v_{s}|/c≲2.5\;\times \;10^{-5}$ per step, two iterations reach machine precision. Equation (4) is the entire coupling: raw signal → time-varying delay → orbit map → epoch state. Its time derivatives carry the Doppler (${\dot{\tau }}_{m}$) and Doppler-rate (${\ddot{\tau }}_{m}$), both large for LEO and both informative. For a non-cooperative emitter the waveform *s* may be unknown; the known-waveform case is developed first for clarity, and the unknown-signal (covariance/QML) generalization is given in Section 6. The principal symbols are collected in Table 1.

## 3. The Direct Estimator

Stacking the received data, the single-step maximum-likelihood (ML) estimate of the orbital state is obtained by concentrating out the unknown per-station complex gains $b_{m}$. Because each $b_{m}$ is an independent nuisance, its ML value at a hypothesized state is ${\hat{b}}_{m}=z_{m}\left(x\right)/E_{s}$, where $z_{m}\left(x\right)=\int y_{m}\left(t\right)\;{\hat{s}}^{*}\left(t-\tau_{m}\left(x,t\right)\right)\;e^{+j2\pi f_{c}\tau_{m}\left(x,t\right)}\;dt$ is the station-*m* matched-filter output, $\hat{s}$ the known (or reconstructed) waveform and $E_{s}$ its energy. Substituting ${\hat{b}}_{m}$ back yields the concentrated objective, which is an *incoherent* (non-coherent) combination across stations. The unknown per-station phases preclude coherent summation:

(5) $$\hat{x}=\underset{x}{\mathrm{arg max}}\;\frac{1}{E_{s}}\sum_{m=1}^{M}|\;\int y_{m}\left(t\right)\;{\hat{s}}^{*}\;\left(t-\tau_{m}\left(x,t\right)\right)\;e^{+j2\pi f_{c}\tau_{m}\left(x,t\right)}\;dt{|}^{2}.$$

(If the relative inter-station gains are known or calibrated, the gain-weighted coherent sum $|\sum_{m}b_{m}^{*}z_{m}{\left(x\right)|}^{2}$ is instead optimal; the conservative unknown-gain case is treated here.), with $\tau_{m}\left(x,t\right)$ given by (4). The key structural point is that (5) is optimized over the six-dimensional epoch state x, and, through (2), every point of the search space is a physically valid orbit. Unlike constrained ground-emitter DPD, where the Earth-surface constraint is imposed separately (and the bound is a constrained CRB [37,38]), here the orbital-dynamics manifold is built into the parameterization: the whole trajectory, i.e., 3N positions for *N* samples, is searched through the six epoch-state parameters regardless of the arc length, and no constraint penalty is required. (Nuisances that cannot be concentrated in closed form, such as the frequency offsets and the emission-time offset of Section 4, add dimensions to the search or are optimized in an inner loop.) Initialization from a coarse two-line element set or a single-pass coarse fix confines the search to a small neighborhood, after which Gauss–Newton refinement converges rapidly (Section 7). The complete procedure is summarized in Algorithm 1.
**Algorithm 1** Direct Orbital-State EstimationThe estimator is implemented in the following steps.1.*Inputs.* Sampled baseband data $y_{m}$ of the *M* stations over the arc; station ECEF coordinates; a common time reference for the stations; the known or structured waveform *s*; a prior epoch state $\bar{x}$ (e.g., propagated from a two-line element set) with a search box of ±Δr per position component and ±Δv per velocity component.2.*Evaluation of the objective for a candidate x.* Propagate ϕ(x,t) over the arc (universal variables, Section 5); rotate the stations to ECI; solve the light-time Equation (4) for every station and sample by two fixed-point iterations; form the matched-filter outputs $z_{m}\left(x\right)$ with the delay and carrier phase compensated; return $\sum_{m}{\left|z_{m}\left(x\right)\right|}^{2}$. Nuisances that cannot be concentrated in closed form ($\delta t_{\mathrm{tx}}$ and, where present, $\delta f_{m}$, ${\dot{f}}_{\mathrm{tx}}$) are maximized in an inner loop. One evaluation costs O(MN) operations, *N* being the number of samples per station.3.*Acquisition.* Maximize the objective over the search box. An exhaustive search at the resolution of the likelihood is impractical (Section 7); the search is organized hierarchically, first by a Doppler-only search on short sub-windows that reduces the box and then over the full arc.4.*Refinement.* Starting from the acquired cell, apply Gauss–Newton (scoring) iterations to (x,η), with the Jacobian obtained from (8) and the closed-form $\Phi_{\;r}$, until the position update is below 1 mm (at most 12 iterations).5.*Output.* The estimate $\hat{x}$ and its predicted covariance, the Schur-complemented CRB of Section 4 evaluated at $\hat{x}$.

The Monte Carlo experiments of Section 7 exercise step 4 (from a single start after acquisition), and the acquisition of step 3 enters through the failure model of the threshold comparison.

## 4. Cramér–Rao Bound

For the deterministic model $\mu_{m}\left(t;x\right)=b_{m}\;s\left(t-\tau_{m}\right)\;e^{-j2\pi f_{c}\tau_{m}}$ in white $\mathrm{CN}(0,N_{0})$ noise, independent across stations, the Fisher information matrix (FIM) is [39]

(6) [J]ij=2N0Re∑m=1M∫∂μm∂xi*∂μm∂xjdt.

This is the conditional FIM for fixed (known) complex gains; the unknown-gain case is handled by the Schur complement of the augmented FIM (nuisance discussion below). The problem reduces to the sensitivity $\partial \mu_{m}/\partial x$, which is built in two layers.

### 4.1. Layer 1: Signal Sensitivity to Its Own Delay

Differentiating $\mu_{m}$ with respect to the scalar delay,

(7) $$g_{m}\left(t\right)\equiv \frac{\partial \mu_{m}}{\partial \tau_{m}}=b_{m}\left[-\dot{s}\left(t-\tau_{m}\right)-j2\pi f_{c}\;s\left(t-\tau_{m}\right)\right]e^{-j2\pi f_{c}\tau_{m}}.$$

The carrier term $-j2\pi f_{c}s$ provides phase-level range sensitivity; the envelope term $-\dot{s}$ provides group-delay (bandwidth) sensitivity. All signal dependence is contained in $g_{m}$.

### 4.2. Layer 2: Delay Sensitivity to the Orbital State

By the chain rule and (4), with $d_{m}\left(t\right)=\phi \left(x,t_{e}\right)-q_{m}\left(t\right)$ evaluated at the retarded epoch $t_{e}=t-\tau_{m}\left(t\right)$ and line-of-sight unit vector ${\hat{u}}_{m}=d_{m}/∥d_{m}∥$, implicit differentiation of the light-time equation gives

(8) $$\frac{\partial \tau_{m}}{\partial x}=\frac{1}{c}\;\frac{{\hat{u}}_{m}{\left(t\right)}^{\;⊤}\;\Phi_{\;r}\left(t_{e},t_{0}\right)}{1+{\hat{u}}_{m}{\left(t\right)}^{\;⊤}v_{s}\left(t_{e}\right)/c}\;\approx \;\frac{1}{c}\;{\hat{u}}_{m}{\left(t\right)}^{\;⊤}\;\Phi_{\;r}\left(t_{e},t_{0}\right),$$

where $\Phi_{\;r}\left(t_{e},t_{0}\right)\in R^{3\times 6}$ is the position block of the orbital state-transition matrix $\Phi (t,t_{0})=\partial \phi /\partial x$, and the denominator, which differs from unity by $|{\hat{u}}_{m}^{\;⊤}v_{s}|/c≲2.5\;\times \;10^{-5}$ for LEO, is retained in the code and dropped in the expressions below for legibility; it changes the bound by less than $10^{-4}$ relative. This is the step that distinguishes the bound from a static-emitter DPD CRB: the line-of-sight sensitivity is projected through the orbital dynamics, so the bound depends on how the entire trajectory responds to a perturbation of the epoch state, not merely instantaneous position.

### 4.3. Assembled FIM

Combining (7) and (8), $\partial \mu_{m}/\partial x=c^{-1}g_{m}\left(t\right)\;{\hat{u}}_{m}{\left(t\right)}^{\;⊤}\Phi_{\;r}\left(t_{e},t_{0}\right)$ with $t_{e}=t-\tau_{m}\left(t\right)$, and (6) becomes

(9) J=2N0c2Re∑m=1M∫Φr⊤(te,t0)u^m(t)|gm(t)|2u^m(t)⊤Φr(te,t0)dt

The integrand factors into three physically distinct contributors:

- *Signal:* $|g_{m}{\left(t\right)|}^{2}={\left|b_{m}\right|}^{2}\left[{\left(2\pi f_{c}\right)}^{2}{\left|s\right|}^{2}+{\left|\dot{s}\right|}^{2}+\mathrm{cross}\right]$, carrying SNR, carrier, and bandwidth—how informative the waveform is per instant;
- *Geometry:* The rank-one dyad ${\hat{u}}_{m}{\hat{u}}_{m}^{\;⊤}$, summed over stations and time, is the classical dilution-of-precision structure;
- *Dynamics:* $\Phi_{\;r}\left(t,t_{0}\right)$, mapping instantaneous position sensitivity back to the epoch state.

The CRB is $C=J^{-1}$; position and velocity RMS bounds are $\sqrt{\mathrm{tr}(C_{rr})}$ and $\sqrt{\mathrm{tr}(C_{vv})}$. The one-line summary: the orbital-state CRB is the multi-station delay CRB projected through the orbital state-transition matrix.

### 4.4. Nuisance Parameters

Unknown complex gains $\left\{b_{m}\right\}$ and station clock offsets are handled by augmenting x and partitioning the FIM, $J=\left[\begin{array}{cc} J_{xx} & J_{x\eta }\\ J_{\eta x} & J_{\eta \eta } \end{array}\right]$, with η the nuisances. The effective orbital-state bound is the Schur complement $C_{xx}={\left(J_{xx}-J_{x\eta }J_{\eta \eta }^{-1}J_{\eta x}\right)}^{-1}$, which quantifies the accuracy loss due to the nuisances. Four classes matter for a non-cooperative satellite, and they enter the two observables differently.

*Constant complex gains* $b_{m}$ affect neither delay nor Doppler geometry and are separable per station. The gain $b_{m}$ is taken constant over the pass; in practice it varies with range, antenna pattern, attitude, and propagation, but, over a single short pass, this variation is neglected in the present analysis, and its impact is left for evaluation as part of the experimental extension.

*Station clock errors* must be split: a constant offset $\delta t_{m}$ contaminates the delay observable, while a clock drift ${\dot{\delta t}}_{m}$ contaminates both delay (linearly in *t*) and, through its rate, the Doppler. Both are per-station and enter as additive terms in the respective observable gradients.

*Emission epoch.* A non-cooperative emitter does not report its transmission time, so each delay also contains the unknown common offset $\delta t_{\mathrm{tx}}$ of Section 2. As with the frequencies, $\delta t_{\mathrm{tx}}$ and the station clock offsets $\delta t_{m}$ appear only through their sums, so they are not separately estimable. If the stations are synchronized to a common reference (e.g., GNSS-disciplined), $\delta t_{m}$ is known and $\delta t_{\mathrm{tx}}$ is a single common nuisance: the delays then contribute time-difference (TDOA) information, and absolute range is lost. If the station clocks are independently unknown, each delay carries its own unknown offset and only its variation over the pass—integrated Doppler—remains, so the delay observable adds essentially no information beyond the Doppler. The results of this paper assume synchronized stations; $\delta t_{\mathrm{tx}}$ enters η and is removed by the Schur complement in the same way as the frequency nuisances. Its effect is largest for small *M*, where the M−1 independent time differences are fewest. For the baseline geometry at ρ=15 dB it raises the three-station position bound from 21 m (emission epoch known) to 41 m, whereas with four stations the bound is 7.0 m in both cases. When the frequency nuisances below are also estimated, the additional cost of $\delta t_{\mathrm{tx}}$ is small (about 76→78 m at M=3) because the Doppler information that remains after the frequency nuisances are removed is then the dominant contribution. All the numerical results of Section 7 include $\delta t_{\mathrm{tx}}$.

**Figure 1:**
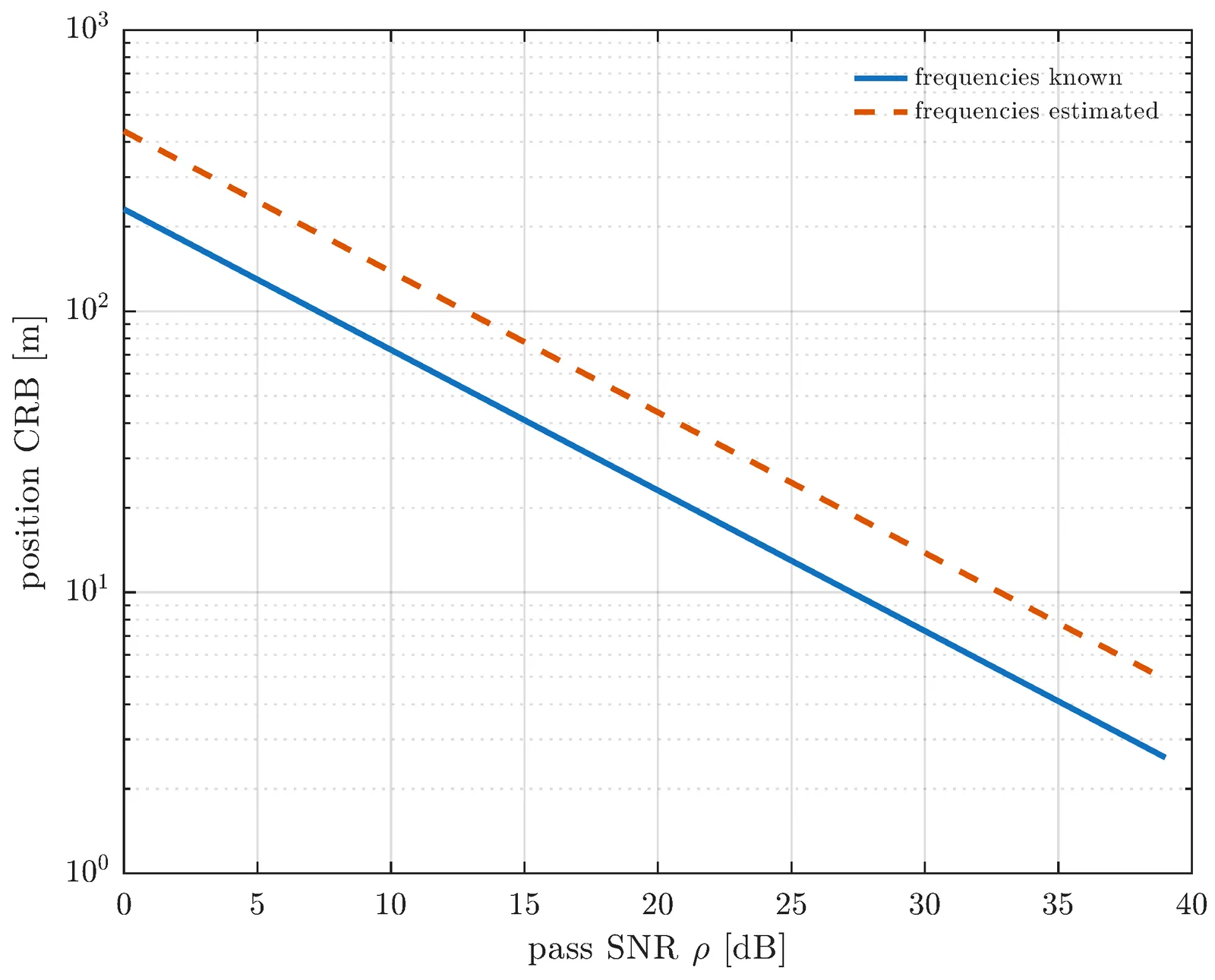
Effect of unknown carrier-frequency offset and drift on the orbital-state CRB. Estimating the identifiable frequency nuisances (per-station offsets and a common drift) jointly with the orbital state via the Schur complement inflates the position CRB by a constant factor (≈1.9) that is independent of SNR. The emission-time offset $\delta t_{\mathrm{tx}}$ is estimated in both curves.

**Figure 2:**
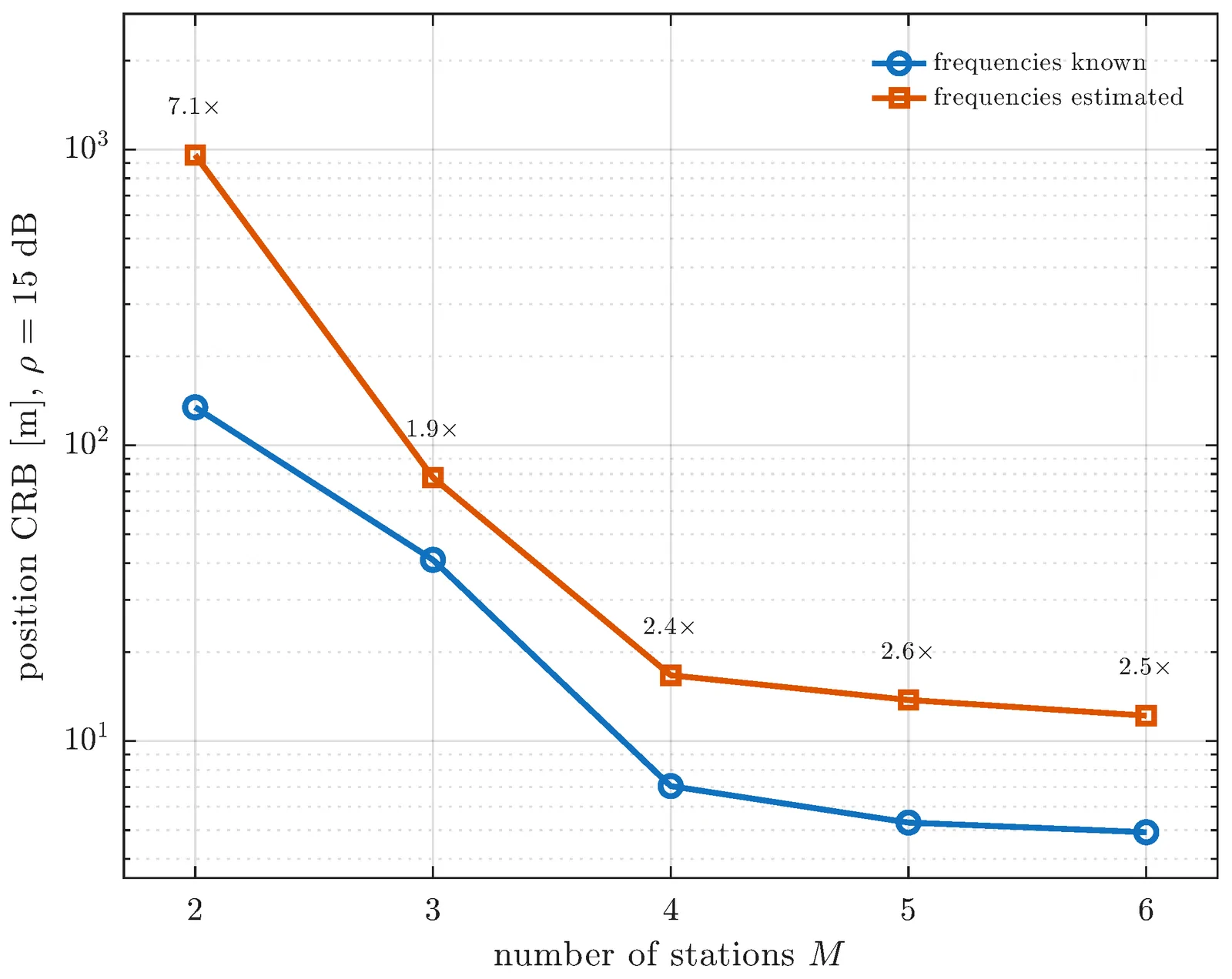
Effective position CRB versus number of stations with frequencies known and the identifiable frequency nuisances estimated (Schur complement). The minimum useful count is unchanged at M=3; the nuisance penalty (annotated) is largest at M=2 (≈7×), and the fourth station gives the largest absolute gain (78→17 m). The emission-time offset is estimated in both curves.

**Figure 3:**
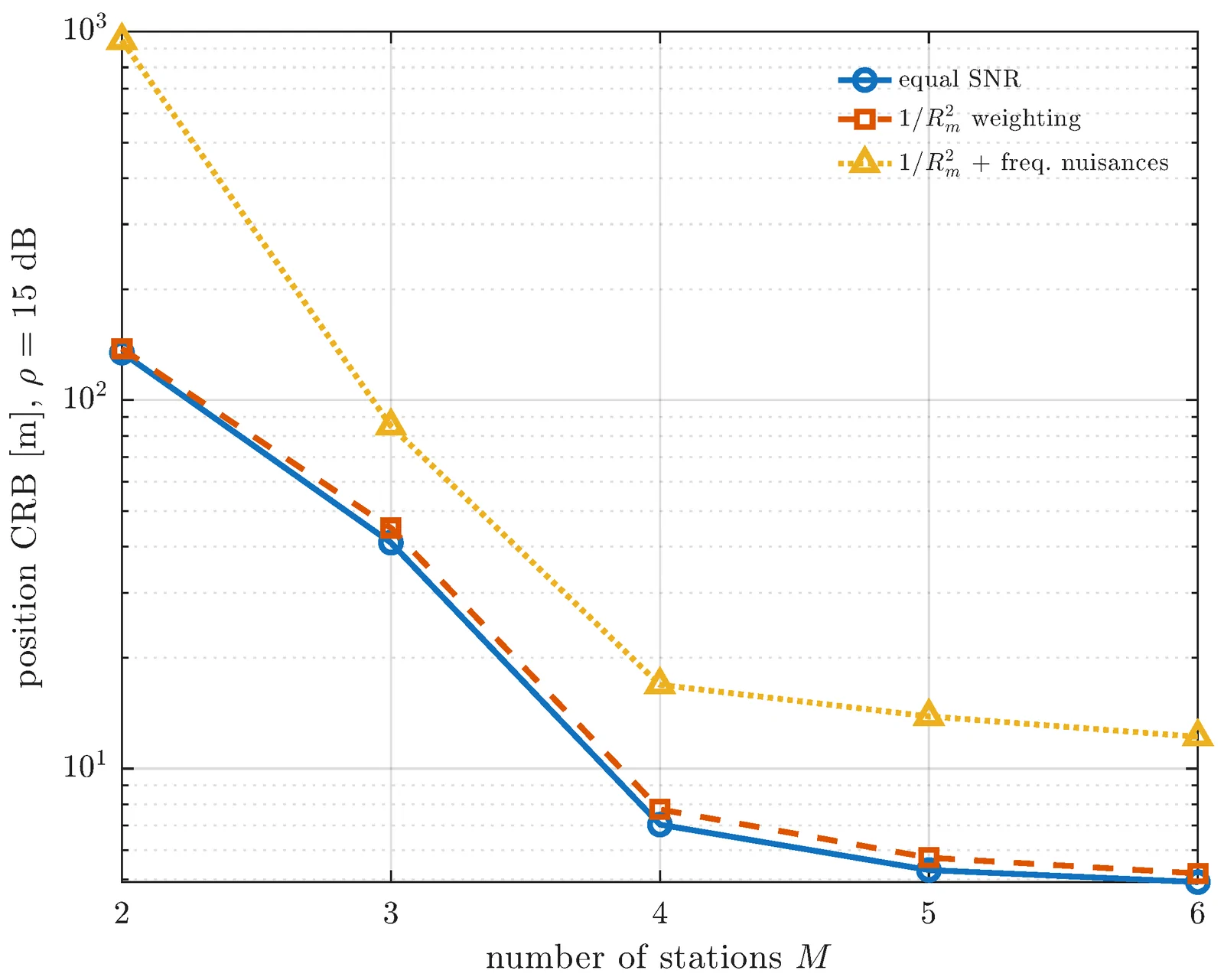
Robustness of the station-count result to range-dependent free-space station weighting. Range-dependent SNR ($|b_{m}{|}^{2}\propto 1/R_{m}^{2}$, fixed total energy) barely shifts the equal-SNR curve; the three/four-station conclusion survives, and M=3 remains usable even with path loss and frequency nuisances combined.

*Carrier-frequency errors* are, for a non-cooperative emitter, the most consequential nuisance and were omitted from earlier treatments. The received Doppler is not $-f_{c}{\dot{\tau }}_{m}\left(t\right)$ alone; it also contains the unknown satellite transmit-frequency offset $\Delta f_{\mathrm{tx}}$ (common to all stations), the transmit oscillator drift ${\dot{f}}_{\mathrm{tx}}$ (common, linear in *t*), and a per-station receiver offset $\Delta f_{m}$. A subtlety of identifiability arises: the common offset $\Delta f_{\mathrm{tx}}$ and the per-station offsets $\Delta f_{m}$ appear only through their sum in each station’s Doppler, so they are not separately estimable; the identifiable set is obtained by eliminating $\Delta f_{\mathrm{tx}}$ and defining the combined per-station offset $\delta f_{m}=\Delta f_{\mathrm{tx}}+\Delta f_{m}$ so that the estimated frequency nuisances are $\left[\delta f_{1},\ldots ,\delta f_{M},{\dot{f}}_{\mathrm{tx}}\right]$. This gauge choice makes the nuisance Fisher block nonsingular by construction rather than relying on an implicit constraint. Appending these to η and taking the Schur complement gives the effective orbital-state CRB in the presence of unknown frequencies. The numerical examples of this subsection (Figure 1, Figure 2 and Figure 3) use the scenario of Section 7: orbit, station coordinates, carrier, bandwidth, arc and observable model are listed there, and the same script produces all the figures. For the baseline geometry, with $\delta t_{\mathrm{tx}}$ estimated, this inflates the position CRB by a constant factor of about 1.9 (41→78 m at 15 dB) across the SNR range (Figure 1): the frequency nuisances tax a fixed fraction of the Doppler information rather than introducing an SNR-dependent floor. Equivalently, a differential formulation—referencing each station’s Doppler to a common one—removes the common terms at the cost of one observable.

The observability and frequency-nuisance analyses can be combined: does the minimum station count change when the identifiable frequency nuisances are estimated jointly with the orbital state? Figure 2 answers this by plotting the effective position CRB versus *M* with and without the frequency nuisances (Schur-complemented). The minimum useful count is unchanged—M=3 still yields a finite fix (with the conditioning quantified in Section 7)—but with all nuisances estimated the bound is large at small *M*: 959 m at M=2, 78 m at M=3, and then 17 m at M=4 and only slowly lower beyond (14 and 12 m at M=5,6). The penalty factor itself is largest at M=2 (≈7×) and lies between 1.9× and 2.6× for M=3–6; the fourth station, which helps to separate the common oscillator drift and per-station offsets from the orbital Doppler, gives the largest absolute gain. The practical implication for the tested regional geometry and pass is that three stations give a finite orbital fix while four or more provide robustness to both geometric conditioning and oscillator (frequency-nuisance) uncertainty; Section 7 shows that the station layout matters as much as the count.

Finally, the equal-SNR assumption is favorable, so range-dependent free-space weighting is also tested. Applying $|b_{m}{|}^{2}\propto 1/R_{m}^{2}\left(t\right)$, with $R_{m}\left(t\right)=∥d_{m}\left(t\right)∥$ the station-to-satellite range, at fixed total received energy (which isolates the effect of redistributing energy across stations and does not test the absolute path-loss penalty), Figure 3 shows the station-count curve is almost unchanged (e.g., 41→45 m at M=3) because the stations lie at comparable ranges to a 543 km orbit, so $1/R_{m}^{2}$ varies little across the network. The station-count result is therefore only weakly sensitive to the redistribution of a fixed total received energy according to free-space range loss, and this holds even when range weighting and the frequency nuisances are combined (M=3 remains finite, *M* ≥ 4 robust).

## 5. Keplerian State-Transition Position Block

The dynamics term $\Phi_{\;r}$ admits a closed form for two-body motion. Universal-variable formulations of Keplerian motion and its state-transition matrix are classical [40,41]; the derivation below obtains the position block directly by implicit differentiation of the universal Kepler equation in a form that reuses the quantities already computed during propagation. Using the Lagrange coefficients, the position is expressed as $r\left(t\right)=f\;r_{0}+g\;v_{0}$, with coefficients f,g that depend on the epoch state, so

(10) $$\begin{array}{cc} \frac{\partial r}{\partial r_{0}} & =f\;I_{3}+r_{0}\;\partial_{r_{0}}f+v_{0}\;\partial_{r_{0}}g, \end{array}$$

(11) $$\begin{array}{cc} \frac{\partial r}{\partial v_{0}} & =g\;I_{3}+r_{0}\;\partial_{v_{0}}f+v_{0}\;\partial_{v_{0}}g, \end{array}$$

and $\Phi_{\;r}=\left[\partial r/\partial r_{0}\;|\;\partial r/\partial v_{0}\right]$. In universal variables with anomaly χ, epoch radius $r_{0}=∥r_{0}∥$, epoch speed $v_{0}=∥v_{0}∥$, epoch radial velocity $v_{r0}=r_{0}^{⊤}v_{0}/r_{0}$, reciprocal semi-major axis $\alpha =2/r_{0}-v_{0}^{2}/\mu$, argument $z=\alpha \chi^{2}$, and Stumpff functions C(z),S(z),

(12) $$f=1-\frac{\chi^{2}}{r_{0}}C\left(z\right),\;g=\Delta t-\frac{\chi^{3}}{\sqrt{\mu }}S\left(z\right).$$

The scalars f,g depend on the epoch state through the primitives $\left\{r_{0},v_{r0},\alpha \right\}$ and χ, which is defined implicitly by the universal Kepler equation $K(\chi ;r_{0},v_{r0},\alpha ,\Delta t)=0$. Implicit differentiation gives

(13) $$\frac{\partial \chi }{\partial x}=-\frac{1}{r(t)}\left(\frac{\partial K}{\partial r_{0}}\frac{\partial r_{0}}{\partial x}+\frac{\partial K}{\partial v_{r0}}\frac{\partial v_{r0}}{\partial x}+\frac{\partial K}{\partial \alpha }\frac{\partial \alpha }{\partial x}\right),$$

where the Jacobian denominator collapses to the instantaneous radius, ∂K/∂χ=r(t)—the defining property $dt/d\chi =r/\sqrt{\mu }$ of the universal anomaly. The primitive gradients are elementary,

(14) $$\frac{\partial r_{0}}{\partial x}=\left[{\hat{r}}_{0}^{⊤}\;\;0^{⊤}\right],\;\frac{\partial \alpha }{\partial x}=\left[\;-\frac{2}{r_{0}^{2}}{\hat{r}}_{0}^{⊤}\;\;-\frac{2}{\mu }v_{0}^{⊤}\right],\;\frac{\partial v_{r0}}{\partial x}=\left[\frac{v_{0}^{⊤}}{r_{0}}-\frac{r_{0}^{\;⊤}v_{0}}{r_{0}^{3}}r_{0}^{⊤}\;\;\frac{r_{0}^{⊤}}{r_{0}}\right],$$

and the coefficient gradients follow with $\partial z/\partial x=\chi^{2}\;\partial \alpha /\partial x+2\alpha \chi \;\partial \chi /\partial x$ and the Stumpff derivatives $C^{\prime }\left(z\right)=\frac{1}{2z}\left(1-zS-2C\right)$, $S^{\prime }\left(z\right)=\frac{1}{2z}\left(C-3S\right)$ (series near z=0). Substituting yields $\Phi_{\;r}$ in closed form, every term a function of the epoch state, χ, and the Stumpff functions—quantities already computed during propagation. For $J_{2}$ or higher perturbations, $\Phi_{\;r}$ is obtained by integrating the variational equations; the CRB structure (9) is unchanged. Classical or equinoctial elements $e_{0}$ are accommodated by the chain rule, $\partial r\left(t\right)/\partial e_{0}=\Phi_{\;r}\;\partial x_{0}/\partial e_{0}$, i.e., by post-multiplying $\Phi_{\;r}$ with the Jacobian of the Cartesian epoch state with respect to the elements.

## 6. Unknown-Waveform Case

When *s* is unknown, the ML estimator (5) is replaced by its quasi-ML/covariance form: the cost is the largest eigenvalue of the cross-station correlation matrix parameterized by x, exactly as in delay–Doppler DPD for unknown signals [4], with the fixed emitter position replaced by the orbital state. It remains useful to establish the corresponding CRB for the unknown-waveform case since it is the conservative non-cooperative model; the estimator-efficiency demonstration of Section 7 uses the known-waveform concentrated ML, and the practical regime of the unknown-waveform case is quantified at the end of this section.

### 6.1. Proposition (Stochastic-Signal FIM)

Model the signal per frequency bin: in each bin the unknown-waveform coefficient is a zero-mean complex-Gaussian scalar of power $\sigma_{s}^{2}$ so that the per-bin spatial covariance is rank-one, $R\left(x\right)=\sigma_{s}^{2}\;a\left(x\right)a{\left(x\right)}^{H}+N_{0}I$, and the total information is the sum over bins, where a(x) is the (frequency-dependent) manifold vector whose parameter dependence is exactly that of the deterministic model. Because the orbital manifold varies over the pass, the snapshots do not share a common a(x): snapshot *ℓ* in bin *n* has its own manifold vector $a_{n\ell }\left(x\right)$, evaluated at the time of that snapshot, and the Slepian–Bangs formula gives the total information as the sum over bins and snapshots, ${\left[J\right]}_{ij}=\sum_{n}\sum_{\ell =1}^{L_{n}}\mathrm{tr}\;\left(R_{n\ell }^{-1}\partial_{i}R_{n\ell }\;R_{n\ell }^{-1}\partial_{j}R_{n\ell }\right)$ with $\partial_{i}R_{n\ell }=\sigma_{s}^{2}\left(\partial_{i}a_{n\ell }\;a_{n\ell }^{H}+a_{n\ell }\;\partial_{i}a_{n\ell }^{H}\right)$. (When the manifold is constant over *L* snapshots the sum collapses to the familiar factor *L*; in the time-varying case the factor is absorbed in the sum, which is why it does not appear explicitly in (15), a per-snapshot expression.) The manifold has one unit-modulus entry per station so that its norm ${∥a\left(x\right)∥}^{2}=M$ is constant in the parameter. Under this convention the rank-one signal covariance reduces, after applying the matrix-inversion lemma, to

(15) [J]ij=2γ2M(1+γ)Re∂iaHPa⊥∂ja,γ=σs2N0∥a∥2,

where $P_{a}^{⊥}=I-aa^{H}/{∥a∥}^{2}$ and γ is the effective array SNR (the per-sample SNR $\rho_{s}=\sigma_{s}^{2}/N_{0}$ scaled by the manifold norm, $\gamma =M\rho_{s}$). The manifold norm is taken constant in the parameter, so no additional amplitude-sensitivity term arises. This is identical in structure to the deterministic FIM (9), the same geometry and dynamics quadratic form $\partial_{i}a^{H}P^{⊥}\partial_{j}a$, which, through (8), carries the ${\hat{u}}_{m}^{⊤}\Phi_{\;r}$ factor with an additional stochastic-signal information factor γ/(1+γ) relative to the corresponding deterministic unknown-amplitude model. That is, relative to the deterministic FIM with the same projected manifold quadratic form Jdet=(2γ/M)Re{∂iaHPa⊥∂ja} (i.e., after the unknown source amplitude has been concentrated out, which is what produces the projector $P_{a}^{⊥}$), the stochastic FIM is reduced by the factor $J_{\mathrm{stoch}}=\frac{\gamma }{1+\gamma }\;J_{\det}$, which approaches unity at high effective array SNR. This relationship refers to the projected unknown-amplitude deterministic FIM, not the conditional known-gain FIM of (9). (Equation (15) was verified numerically against the deterministic FIM; the ratio matches γ/(1+γ) to four significant figures across γ∈[−10,20] dB).

### 6.2. Practical Regime of the Unknown-Waveform Case

The factor γ/(1+γ) is innocuous only when the *per-snapshot* array SNR γ is not small, and, for a wideband long capture, it is very small: with N=BT samples per station and pass SNR ρ, the per-sample SNR is $\rho_{s}=\rho /N$ and $\gamma =M\rho_{s}$. For the scenario of Section 7 (B=2 MHz, T=60 s, $N\approx 1.2\times 10^{8}$, M=3) a pass SNR of 15 dB corresponds to $\rho_{s}\approx -66$ dB and $\gamma \approx 8\times 10^{-7}$, so the unknown-waveform bound is about $10^{3}$ times the deterministic one, and cross-station correlation of a Gaussian waveform is above its detection threshold only when $N\rho_{s}^{2}\gg 1$, i.e., for pass SNRs of the order of 45–50 dB. The unknown-waveform direct estimator is therefore viable for strong narrowband or short captures (high $\rho_{s}$), whereas, at low-pass SNRs where the single-step advantage of Section 7 is predicted, the waveform must be known or at least structured (a known modulation format, a pilot, or a cyclostationary signature), which is the usual situation for a non-cooperative but standard-compliant downlink. The low-SNR results of this paper are accordingly stated for the known-waveform model, and the extension of the efficiency demonstration to structured-waveform estimators is left to future work.

The numerical non-cooperative signal model retains delay/Doppler geometry and does not exploit carrier-phase ranging. Although the signal model (3) contains the carrier-phase term $e^{-j2\pi f_{c}\tau_{m}\left(t\right)}$, the unknown constant complex gain $b_{m}$ absorbs the absolute per-station carrier phase; the estimator therefore exploits the *time-varying* phase evolution (Doppler and Doppler rate), not absolute carrier-phase ranging. This is what the “delay/Doppler” description of the numerical observable model (Section 7) refers to.

## 7. Numerical Results

Table 2 collects the modeling assumptions, which are layered: a core idealized model, the two signal (waveform) models, the nuisance model, and the numerical scenario.

**Figure 4:**
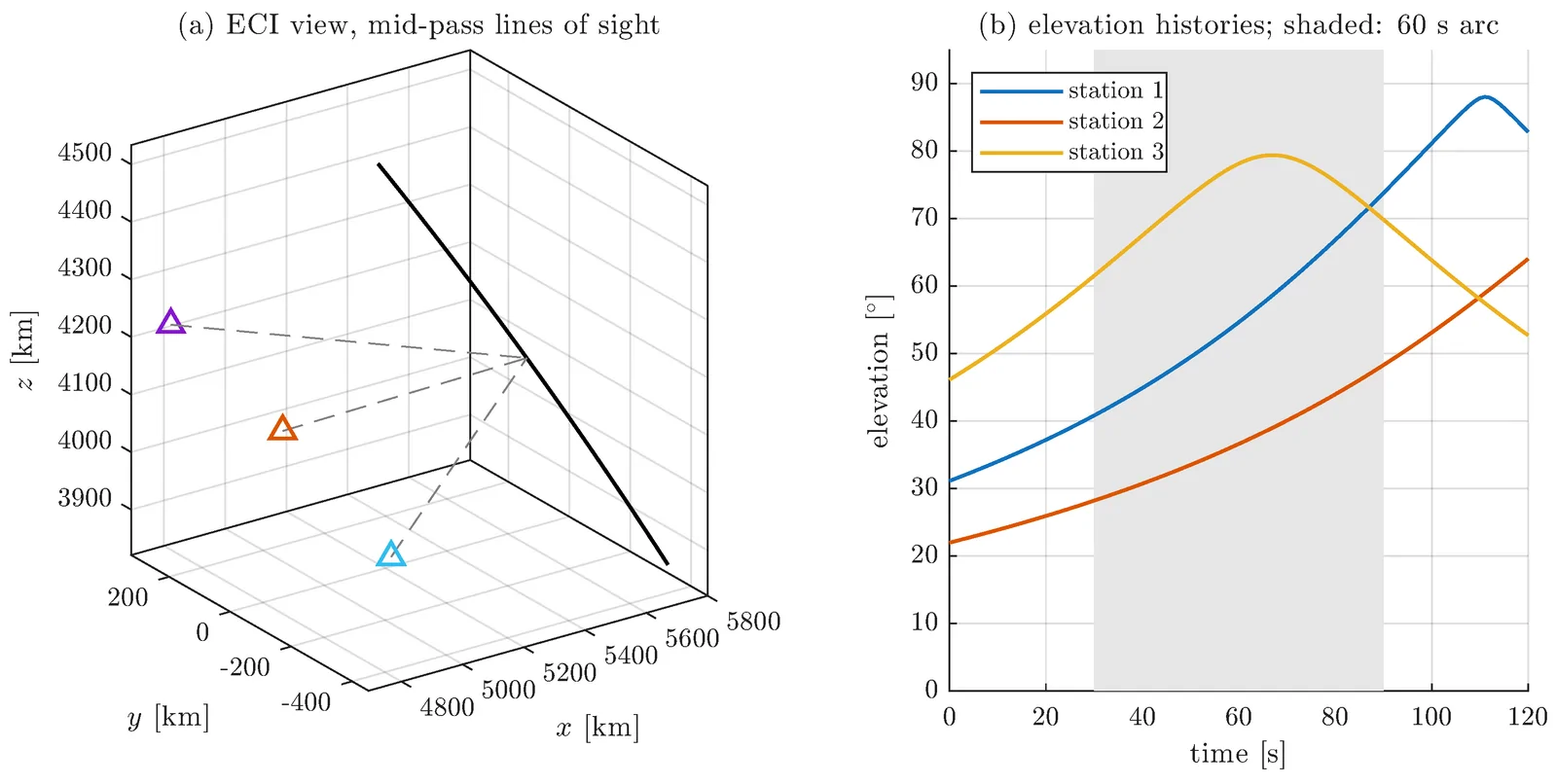
Observing geometry. (**a**) Earth-centered inertial (ECI) view of the LEO pass (solid), the three ground stations (triangles, in the station colors of panel (**b**)), and the station-to-satellite lines of sight at mid-pass (dashed). (**b**) Elevation angle of the satellite from each station over the pass; the staggered peaks provide the geometric diversity that conditions the Fisher information. Inter-station baselines are 303–611 km.

Table 3 maps each numerical experiment to the signal model, the nuisance assumptions and the bound against which it is compared; the observing geometry common to all the experiments is shown in Figure 4.

### 7.1. Scenario

**Figure 5:**
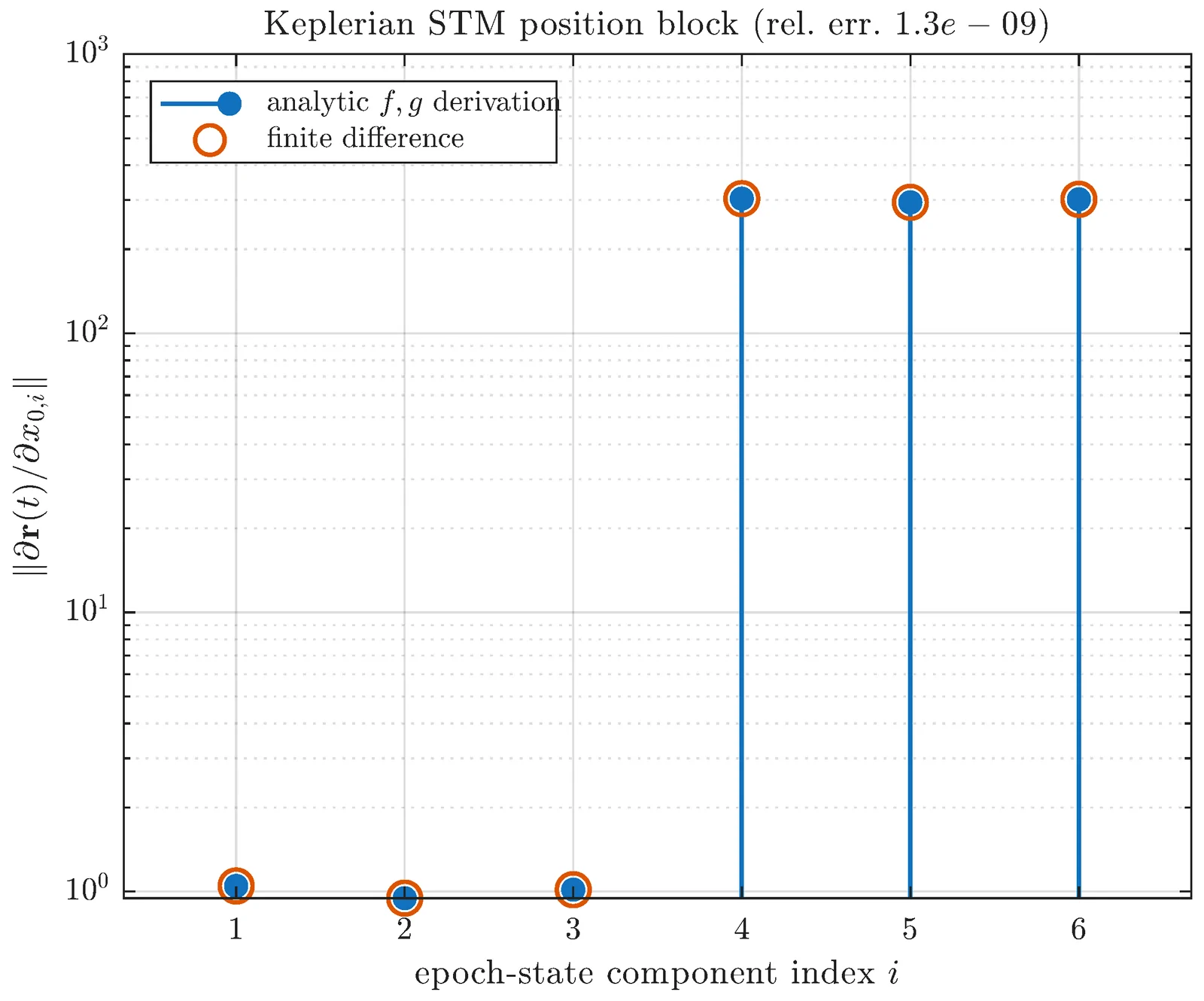
Analytic vs. finite-difference position block of the Keplerian state-transition matrix; column norms coincide to relative error $∼\;10^{-9}$ (finite-difference steps of 1 m and $10^{-3}$ m/s).

**Figure 6:**
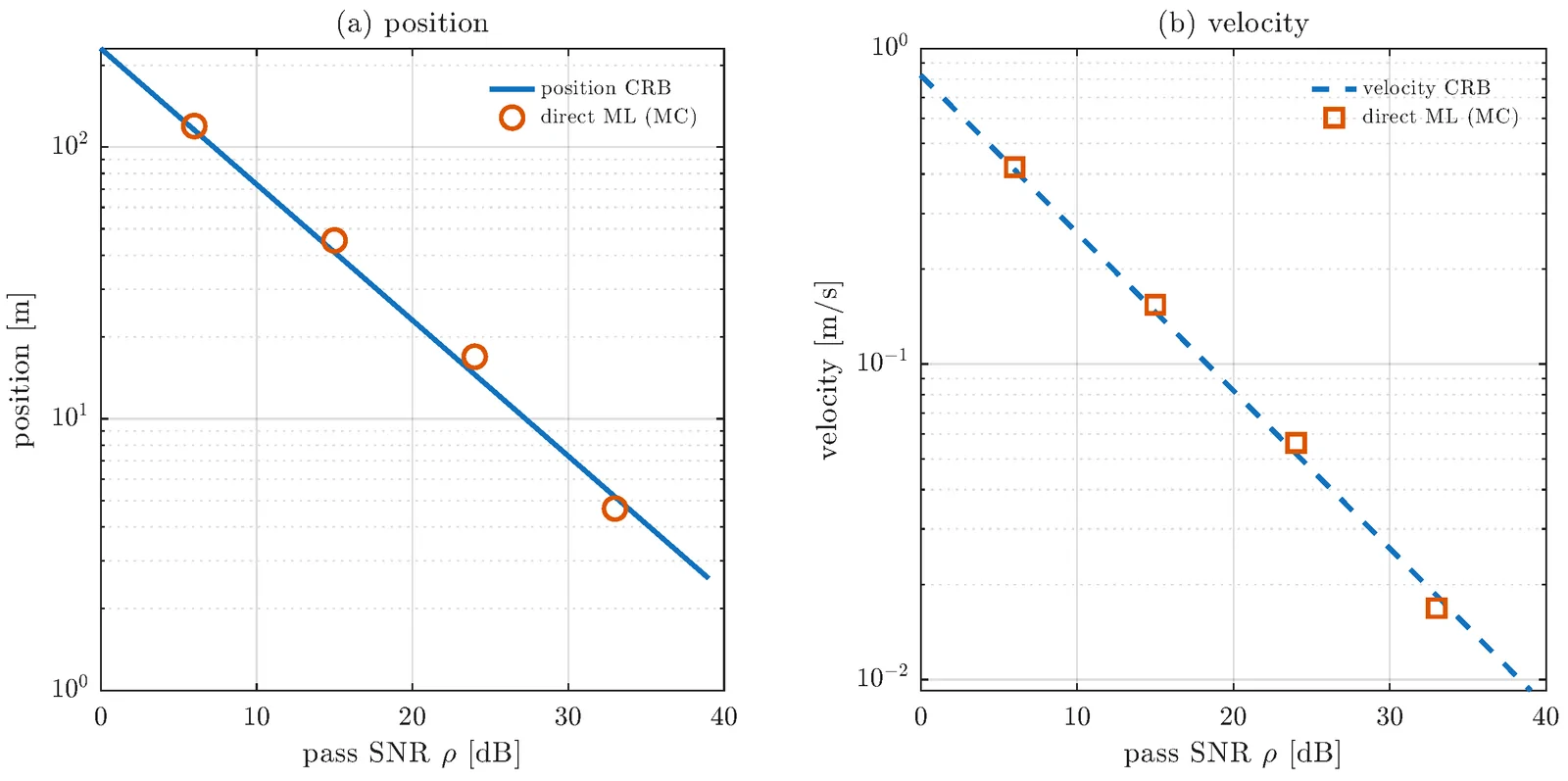
Orbital-state CRB versus pass SNR, shown as (**a**) position and (**b**) velocity on separate axes. The direct known-waveform estimator, simulated after acquisition under the Gaussian delay/Doppler surrogate (Monte Carlo markers), attains both bounds. The ratio $\sigma_{r}/\sigma_{v}\approx 280$ s (an effective dynamic timescale) is constant in SNR; the panels are separated so this fixed offset is not read as overlap.

**Figure 7:**
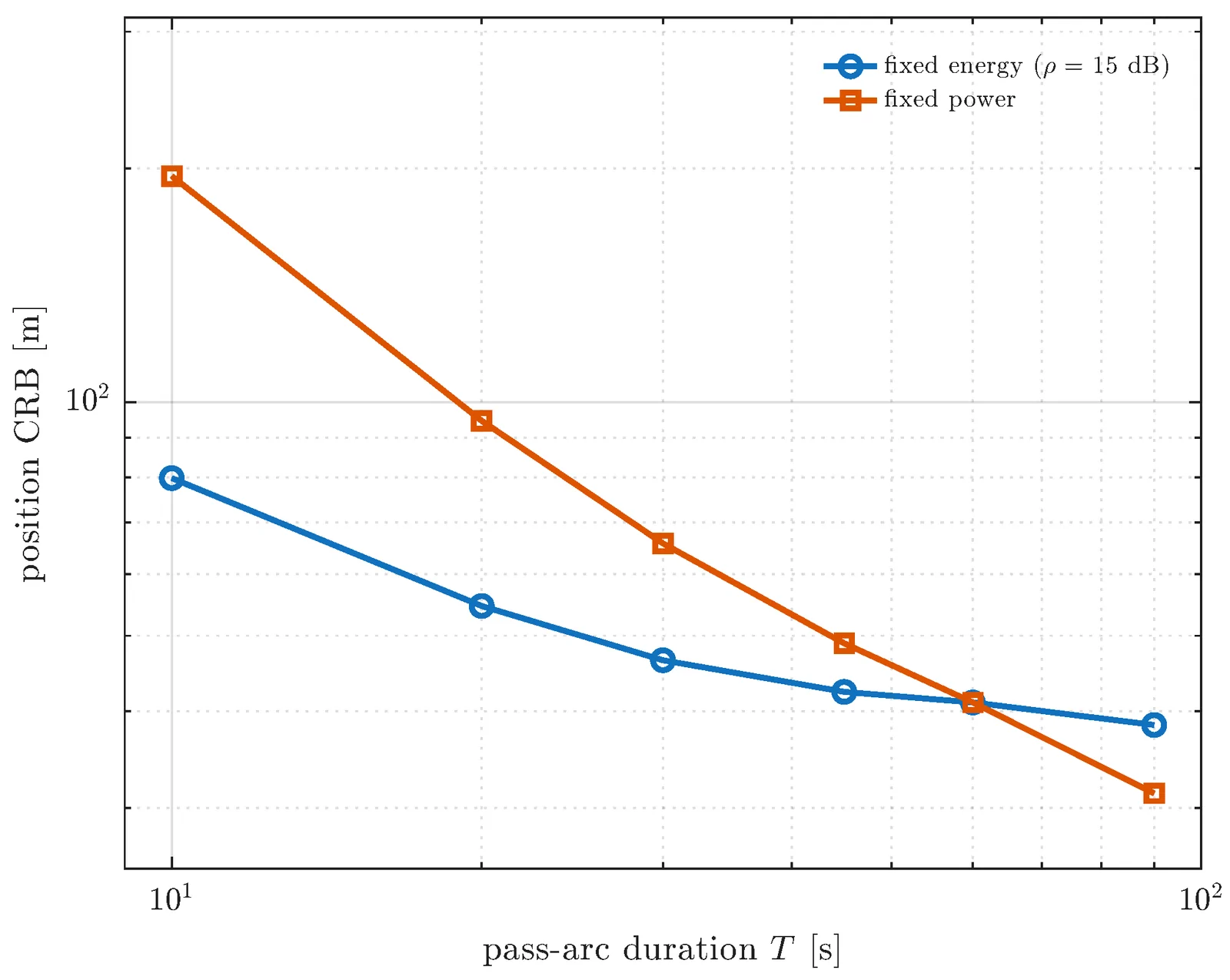
Position CRB versus pass-arc duration under two normalizations: fixed total pass energy (ρ=15 dB held constant as *T* grows) and fixed received power (ρ growing with *T*). The fixed-energy curve isolates the information gained purely from changing geometry and dynamics, most of it within the first 30 s; the fixed-power curve adds the ordinary integration gain.

The observing geometry is shown in Figure 4. A near-circular LEO orbit at 543 km altitude and inclination $65^{\circ }$ is observed by M=3 ground stations on Earth’s surface, at $\left(40^{\circ },0^{\circ }\right)$, $\left(41.5^{\circ },3^{\circ }\right)$, and $\left(38^{\circ },-2.5^{\circ }\right)$ latitude/longitude, forming a regional network with inter-station baselines of 303 km, 310 km, and 611 km. The satellite transits the network over a 120 s window during which all three stations observe it above a $10^{\circ }$ elevation mask (elevations from $22^{\circ }$ to $88^{\circ }$) with complementary elevation histories (Figure 4b): each station reaches peak elevation at a different time, providing the line-of-sight diversity that conditions the estimation problem. All the results of this section were produced by one script from this pass (sub-satellite point $34^{\circ }$ N, $4^{\circ }$ W at the epoch $t_{0}=0$, heading along the $65^{\circ }$-inclination track, 60 s arc starting at t=30 s) with the light-time delay model. The carrier is $f_{c}=2.2$ GHz and the rms bandwidth B=2 MHz; the pass-arc duration is 60 s unless varied. The scenario data of Figure 5, Figure 6 and Figure 7 are those of this section: the same orbit (543 km, $65^{\circ }$), stations, carrier, bandwidth and arc; the STM validation of Figure 5 uses the same epoch state propagated over Δt=300 s. All the numerical results include Earth rotation: station positions and velocities are transformed into the ECI frame as functions of time, and the station velocity is retained in the Doppler. Unless otherwise stated, the numerical CRBs below use the ideal frequency-known model; the carrier-frequency nuisance penalty is reported separately in Figure 1. All the results assume synchronized stations and estimate the common emission-time offset $\delta t_{\mathrm{tx}}$ of Section 4 jointly with the state (Schur complement for the bounds, an additional unknown in the estimators), so the delays contribute time-difference information. The numerical observable model is a delay/Doppler surrogate for a known or sufficiently structured non-cooperative waveform. The unstructured unknown-Gaussian-waveform case of Section 6 is treated analytically only.

### 7.2. Numerical Observable Model

The numerical bounds are computed for a windowed delay/Doppler surrogate of (9). Each station’s pass is divided into $N_{s}$ samples; at each sample the delay and the Doppler are treated as independent Gaussian observables with per-sample information $2\rho_{s}{\left(2\pi B\right)}^{2}$ and $2\rho_{s}{\left(2\pi T_{d}\right)}^{2}$, i.e., variances

(16) $$\sigma_{\tau }^{2}=\frac{1}{2\rho_{s}{\left(2\pi B\right)}^{2}},\;\sigma_{f}^{2}=\frac{1}{2\rho_{s}{\left(2\pi T_{d}\right)}^{2}},$$

where $\rho_{s}=\rho /N_{s}$ is the per-sample SNR, *B* the rms bandwidth and $T_{d}$ the effective rms duration of the window that produces each Doppler observable ($T_{d}$ plays the role of *B* for frequency; $\sigma_{f}$ is in Hz). The value $T_{d}=1$ s is used throughout. Because the per-sample information scales as $1/N_{s}$, the total information does not depend on $N_{s}$. The surrogate treats the Doppler of successive windows as independent and so does not exploit phase continuity across windows; it is therefore conservative relative to the full-data information (9), which retains the coherent phase evolution over the arc.

### 7.3. STM Validation

Figure 5 validates the closed-form Keplerian state-transition position block of Section 5. Each of the six abscissae is one component of the epoch state x: indices 1–3 are the epoch position $r_{0}$ and indices 4–6 the epoch velocity $v_{0}$. The ordinate is the Euclidean norm of the corresponding column of $\Phi_{\;r}\left(t,t_{0}\right)$, i.e., $∥\partial p_{s}\left(t\right)/\partial x_{0,i}∥$—the sensitivity of the satellite position at time *t* to a unit perturbation of that epoch-state component. Two features are physically meaningful and both are reproduced exactly by the analytic expression: the velocity-sensitivity block (indices 4–6) has a characteristic scale of order Δt (here 300 s), as expected from first-order kinematic propagation, whereas the position-sensitivity block (indices 1–3) is O(1). This comparison is unit-dependent (the position-state sensitivity $\partial r/\partial r_{0}$ is dimensionless, while the velocity-state sensitivity $\partial r/\partial v_{0}$ has units of time), so the raw column magnitudes should not be compared without normalization by representative state scales. Over the propagation interval (here Δt=300 s), the velocity-state sensitivity scales roughly as Δt. The analytic f,g derivation and an independent central finite difference of the propagated position agree to a relative error $∥\Phi_{\;r}^{\mathrm{an}}-\Phi_{\;r}^{\mathrm{fd}}{∥}_{F}/{∥\Phi_{\;r}^{\mathrm{fd}}∥}_{F}∼10^{-9}$, the finite difference being taken with fixed steps of 1 m in each epoch-position component and $10^{-3}$ m/s in each epoch-velocity component, the propagation itself being the closed-form universal-variable solution (no ODE solver is involved; MATLAB R2025b, MathWorks, Natick, MA, USA), providing a numerical verification of the closed-form result. The finite-difference check requires no orbital-mechanics software and is the recommended verification before the bound is trusted.

### 7.4. CRB and Efficiency

Figure 6 shows the position (panel a) and velocity (panel b) CRB versus pass SNR on separate axes. Here the pass SNR ρ is defined as the total received signal-energy-to-noise ratio per station, integrated over the observation interval; per-sample SNR is $\rho /N_{s}$ for $N_{s}$ samples. The stations are taken to have equal SNRs (identical $|b_{m}|$) in the numerical scenario; this is a favorable choice since the stations span different ranges and elevation histories. A range-dependent (free-space path-loss) SNR would weight the stations unequally; its effect on the station-count result is quantified in Figure 3 and found to be small. Both bounds fall as SNR^−1/2^ (an exact −1 dB/dB slope): 41 m and 0.15 m/s at ρ=15 dB, reaching meter-level position and cm/s velocity accuracy at the upper end of the range for a single pass. Because the two bounds share this slope, their ratio is *constant in SNR* (here $\sigma_{r}/\sigma_{v}\approx \;280$ s, an effective dynamic timescale set by the geometry and propagation interval); they are plotted separately so this fixed offset is not mistaken for coincidence. Monte Carlo runs of the direct estimator (Gauss–Newton on the delay/Doppler geometric parameters, circles and squares) lie on both bounds. This estimator is the ML estimator under the Gaussian delay/Doppler surrogate of the known-waveform model, used here as a tractable efficiency demonstration. Section 6 gives the corresponding stochastic (unknown-waveform) FIM, which differs from the corresponding projected deterministic FIM by the scalar γ/(1+γ) (a relation between those two manifold models, not a correction to be applied to the surrogate bound plotted here); demonstrating QML efficiency against that bound is left for a dedicated unknown-waveform Monte Carlo study.

### 7.5. Arc Length

Figure 7 shows the position CRB versus pass-arc duration. Accuracy improves rapidly with the observed orbital arc: a longer arc both sweeps the line of sight over more directions and exposes more of the orbit’s dynamic response, tightening $\Phi_{\;r}$ in (9). To separate the sources of this improvement, Figure 7 plots the position CRB versus arc under two normalizations: fixed total pass energy (holding ρ constant as *T* grows) and fixed received power (letting the integrated energy grow with *T*). The fixed-power curve improves steadily, as expected from the added integration gain. The fixed-energy curve is the more interesting one: with no additional received energy the bound still falls from 80 m at 10 s to 47 m at 30 s, 42 m at 45 s and 38 m at 90 s because lengthening the observation changes the line-of-sight geometry and the state-transition sensitivity; most of the gain is obtained within the first 30 s, after which the improvement is slow. Thus, at fixed total received energy, orbital motion increases the information about the epoch state through the time evolution of the observation manifold ${\hat{u}}_{m}^{⊤}\left(t\right)\Phi_{\;r}\left(t,t_{0}\right)$—a feature of a moving orbital emitter that is absent from static-emitter DPD—but the gain is bounded and geometry-dependent; the integration gain of the fixed-power curve is what makes long arcs pay.

### 7.6. Station Geometry

A single station yields a numerically rank-deficient Fisher information for the measurement model, arc and geometry studied here: the geometry factor ${\hat{u}}_{m}{\hat{u}}_{m}^{⊤}$ in (9) is then a sum of nearly collinear rank-one dyads whose time-integrated contribution produces an effectively rank-deficient Fisher information for the tested geometry. Each additional suitably separated station—observing the pass from a distinct line-of-sight history (Figure 4b)—fills this factor and conditions ***J***, sharply reducing the bound. In the three-station geometry of Figure 4 the Fisher information has full rank throughout the pass; its conditioning is quantified below.

**Figure 8:**
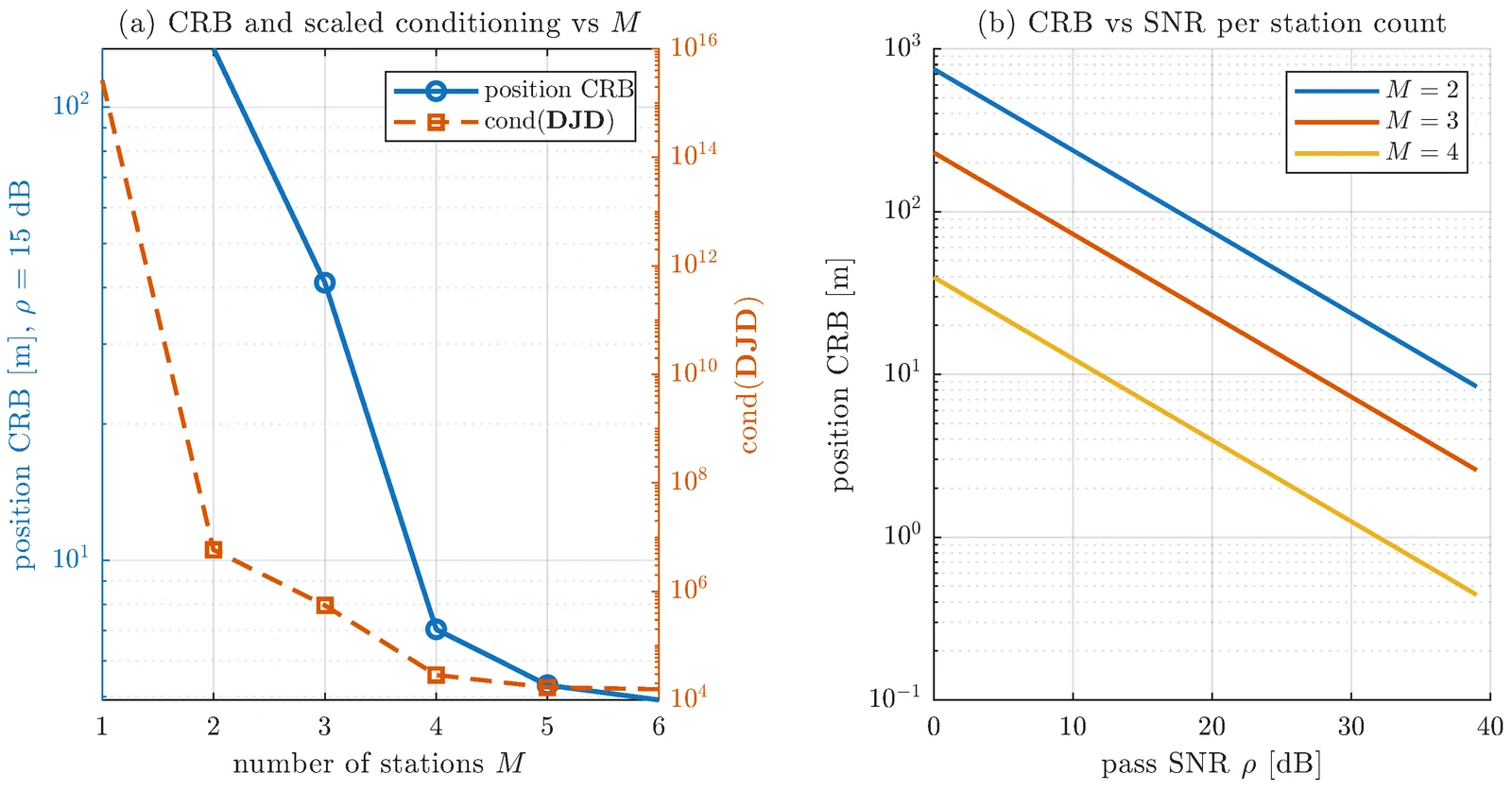
Accuracy vs. number of ground stations. (**a**) Position CRB (left axis) and condition number of the nondimensionalized information (right axis) vs. *M* at *ρ* = 15 dB; shaded bands mark the observability-limited (*M* ≲ 4) and SNR-limited (*M* ≳ 4) regimes. (**b**) Position CRB vs. SNR for *M* = 2, 3, 4; a single station leaves the state unobservable.

Figure 8 quantifies this dependence. Panel (a) shows the position CRB and the scaled FIM condition number as stations are added from a regional pool of six; panel (b) shows the CRB versus SNR for each station count. Two regimes appear. With one or two stations the Fisher information is rank-deficient or nearly so and the state is effectively unobservable, so each added station lowers the bound by orders of magnitude—an observability-limited regime driven by geometric diversity, not SNR. Beyond about four stations further stations give the ordinary $O(M^{-1/2})$ improvement—an SNR-limited regime. Three stations produce a full-rank FIM in this geometry; four or more improve the conditioning. Because the state mixes position and velocity, the condition number and the singular values of ***J*** in SI units depend on the choice of units; the observability statements below are therefore made on the nondimensionalized information $\tilde{J}=DJD,\;D=\mathrm{diag}(a\;I_{3},\;v_{\mathrm{circ}}I_{3})$ with *a* the semi-major axis and $v_{\mathrm{circ}}$ the circular speed as characteristic scales (a Jacobi scaling by ${\mathrm{diag}(J)}^{-1/2}$ gives the same qualitative picture), and exact rank deficiency is distinguished from numerical conditioning. The transition identifies the minimum network size that yields an observable well-conditioned orbital fix for the tested geometry.

**Figure 9:**
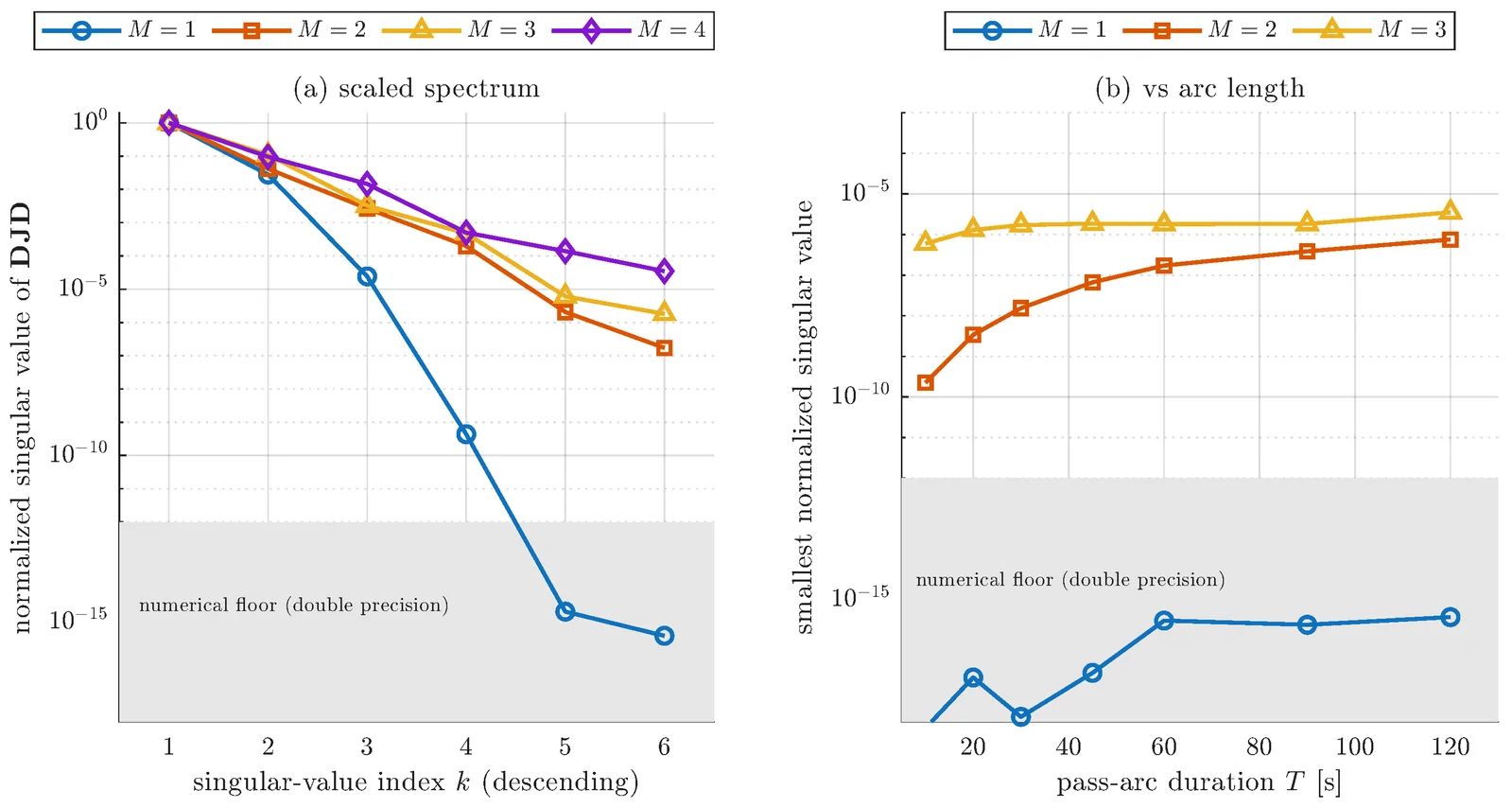
Observability from the time-integrated Fisher information. (**a**) Normalized singular values of the nondimensionalized information $\tilde{J}$ (state scaled by *a* and *v*_circ_ for *M* = 1, …, 4 in descending order of the index *k*; each index is a direction (singular vector) in the scaled state space, a combination of position and velocity components rather than a single component. Shaded: below 10^−12^, numerically unobservable. (**b**) Smallest normalized singular value versus pass-arc duration for *M* = 1, 2, 3.

Because the line-of-sight direction rotates during a pass, rank deficiency cannot be inferred from the instantaneous geometry factor ${\hat{u}}_{m}{\hat{u}}_{m}^{⊤}$ alone; it must be established from the time-integrated Fisher information. Figure 9a shows the six singular values of the scaled information $\tilde{J}$ for *M* = 1, …, 4: a single station leaves two modes far below the numerical floor (numerical rank deficiency for this measurement model, arc and geometry; the statement is not a generic single-station Doppler result), two stations leave one marginal mode (smallest normalized singular value ~10^−7^), and three stations give full rank with a scaled condition number of about 5 × 10^5^, reduced by more than an order of magnitude (to 3 × 10^4^) with a fourth station. Figure 9b confirms this is not a short-arc artifact: the smallest scaled singular value for *M* = 1 remains below 10^−12^ across the full range of pass durations, so the rotation of the line of sight does not by itself restore single-station observability; for *M* = 2 and 3 it grows with the arc.

### 7.7. Station Layout and Pass

**Figure 10:**
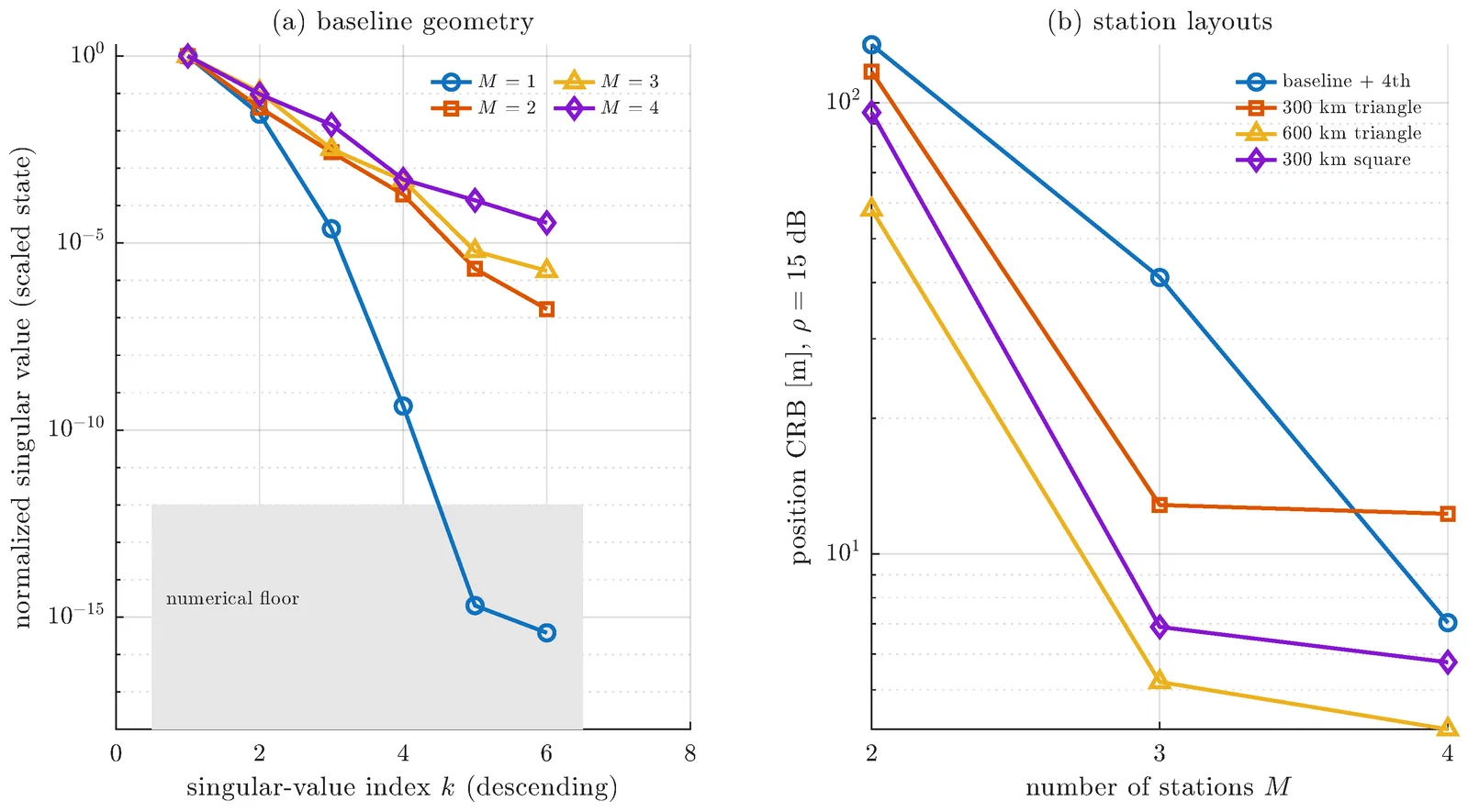
Sensitivity of observability to the station layout. (**a**) Normalized singular values of the nondimensionalized information for M=1,…,4 on the baseline layout plus a fourth station, indexed as in Figure 9a. (**b**) Position CRB at ρ=15 dB versus station count for four layouts: the near-collinear baseline with a fourth station added, equilateral triangles of 300 and 600 km sides with a fourth station at their center, and a 300 km square.

The three stations of Figure 4 lie nearly on a line, which is the least favorable layout for a given count. Figure 10 repeats the observability and bound analysis for an equilateral triangle of 300 km side, an equilateral triangle of 600 km side, and a 300 km square with four stations on the same pass. At M=3 and ρ=15 dB the position bound is 41 m for the near-collinear layout, 13 m for the 300 km triangle and 5 m for the 600 km triangle; the square gives 7 m with three of its stations and 6 m with all four, and a fourth station at the center of either triangle adds comparatively little (12.8→12.3 and 5.2→4.1 m) because it repeats lines of sight the vertices already provide. The unknown emission epoch costs most in the near-collinear layout (21→41 m) and little in the well-separated ones. The scaled condition number improves correspondingly, from $5\times 10^{5}$ to $5\times 10^{4}$ and $9\times 10^{3}$. The single-station rank deficiency (two modes below the numerical floor) and the marginal two-station mode (smallest normalized singular value ∼$10^{-7}$) are the same in every layout. Two further passes over the baseline network (a descending pass and a north-westward pass, not shown) give three-station bounds of 18 and 23 m, so the baseline pass of Figure 4 is the least favorable of the three and the three-station results lie within a factor of about 2.3 of one another; with the emission epoch known the spread would be within a factor of 1.5 (15–22 m). Well-separated non-collinear stations are therefore worth as much as an additional station, which is the familiar dilution-of-precision conclusion; the count statements above are for the tested regional layouts and are not universal.

### 7.8. Light-Time Correction

With the retarded-epoch delay model (4) the bounds of Figure 6, Figure 7, Figure 8, Figure 9 and Figure 10 change by less than $10^{-4}$ relative to the simultaneous-time approximation because the sensitivity (8) differs only by the factor $1/(1+{\hat{u}}_{m}^{⊤}v_{s}/c)$ and by the evaluation of the line of sight and of the transition matrix at the retarded epoch.

The approximation is nevertheless not admissible for the estimator: fitting the simultaneous-time model to noise-free observables generated with the light-time model on the reproduced pass leaves a pseudo-true bias of 276 m in position (27 m along track) and 1.04 m/s in velocity—more than six times the 15 dB bound and far above the meter-level bounds at high SNR. All the estimator results in this paper use the light-time model; the light time at the start of the arc is 2.6 ms, during which the satellite moves 20 m.

### 7.9. Estimator Validation

To confirm that the CRB is operationally meaningful—not merely an asymptotic limit—the direct estimator is exercised in a Monte Carlo experiment under the Gaussian delay/Doppler surrogate of (16). In each trial, and for every station and every one of the 60 time samples of the arc, a delay and a Doppler measurement are drawn from the Gaussian distributions (16) about the light-time-model values, with a common unknown offset $\delta t_{\mathrm{tx}}$ added to all delays; the six-dimensional orbital state and $\delta t_{\mathrm{tx}}$ are then estimated jointly by weighted Gauss–Newton (at most 12 iterations) from a state perturbed by 50 m and 0.5 m/s per component. This is the ML estimator under the surrogate model, which stands in for the concentrated ML of Equation (5) after acquisition, and it exercises the geometry and dynamics layers of the bound, the light-time model and the solver; it does not simulate the raw-sample likelihood surface or its acquisition (see below). Across the signal-to-noise range investigated the post-acquisition root-mean-square error is consistent with the deterministic CRB within the Monte Carlo uncertainty of 15–30 trials per point (Figure 6): 120, 45, 17 and 4.7 m at 6, 15, 24 and 33 dB against bounds of 115, 41, 14.5 and 5.2 m with no gross errors. This also serves as a consistency check on the derivation.

*Acquisition.* The Monte Carlo starts after acquisition. A raw-sample search of the direct likelihood over a prior box of the size of a two-line-element error, ±2 km in each position component and ±2 m/s in each velocity component, at the resolution of the likelihood (about c/B=150 m in range and λ/T≈2 mm/s in radial velocity, with $\lambda =c/f_{c}$ the wavelength, for a 60 s coherent arc) comprises $N_{\mathrm{cell}}^{\mathrm{dir}}\approx 10^{14}$ cells and is not practical by exhaustive search; in practice it has to be organized hierarchically—a coarse Doppler-only search on short sub-windows followed by refinement—as in the acquisition stage of the two-step chain. Because the surrogate likelihood used in the Monte Carlo is unimodal, it cannot produce acquisition errors by itself; the next paragraph therefore models the acquisition failures of both estimators explicitly with the same independent-cell argument.

### 7.10. Model-Based Threshold Comparison with Two-Step Processing

A two-step estimator first extracts, per station and per sub-window, a delay and a Doppler observable from the received data, then fits the orbital state to those observables. Above a signal-to-noise threshold this is asymptotically equivalent to the direct estimator and attains the same CRB. Below threshold, however, the per-sub-window observable extraction—a peak search over the delay/Doppler cross-ambiguity surface—begins to select noise peaks, injecting gross outliers that the orbit fit cannot repair. This effect is quantified here for both estimators with a model of the wrong-peak probability rather than a raw-sample simulation; the threshold locations are derived as follows. Within each sub-window of duration $T_{\mathrm{sub}}=T/K$, the two-step estimator locates a delay and a Doppler by searching the cross-ambiguity function (CAF) over an a priori (coarse-ephemeris) box of extent Δτ in delay and Δf in Doppler. The CAF is sampled at its resolution, 1/B in delay and $1/T_{\mathrm{sub}}$ in Doppler. The independent-cell approximation used below applies when cells are sampled at approximately the ambiguity resolution; residual cell correlation can be accounted for approximately through an effective number of independent cells. The number of cells searched is

(17) $$N_{\mathrm{cell}}=\left(\Delta \tau \;B\right)\left(\Delta f\;T_{\mathrm{sub}}\right).$$

For a matched CAF [7] in complex Gaussian noise, each non-signal cell has an exponentially distributed magnitude-squared, so a single noise cell exceeds the signal cell with probability $e^{-\rho_{\mathrm{sub}}}$, where $\rho_{\mathrm{sub}}=\rho /K$ is the per-sub-window SNR. The probability that the peak is picked incorrectly is, under a high-SNR (fixed-signal-cell) approximation in which the signal-cell statistic is treated as deterministic,

(18) $$P_{\mathrm{wrong}}=1-{\left(1-e^{-\rho_{\mathrm{sub}}}\right)}^{N_{\mathrm{cell}}-1}\;\approx \;N_{\mathrm{cell}}\;e^{-\rho_{\mathrm{sub}}},$$

which is the outlier mechanism analyzed for frequency estimation in [42] and, for maximum-likelihood estimators in general, in [43,44]. It crosses order unity at the per-sub-window threshold $\rho_{\mathrm{sub}}^{★}=\ln N_{\mathrm{cell}}$, i.e., at a total-pass threshold

(19) $$\rho^{★}=K\;\ln N_{\mathrm{cell}}.$$

**Figure 11:**
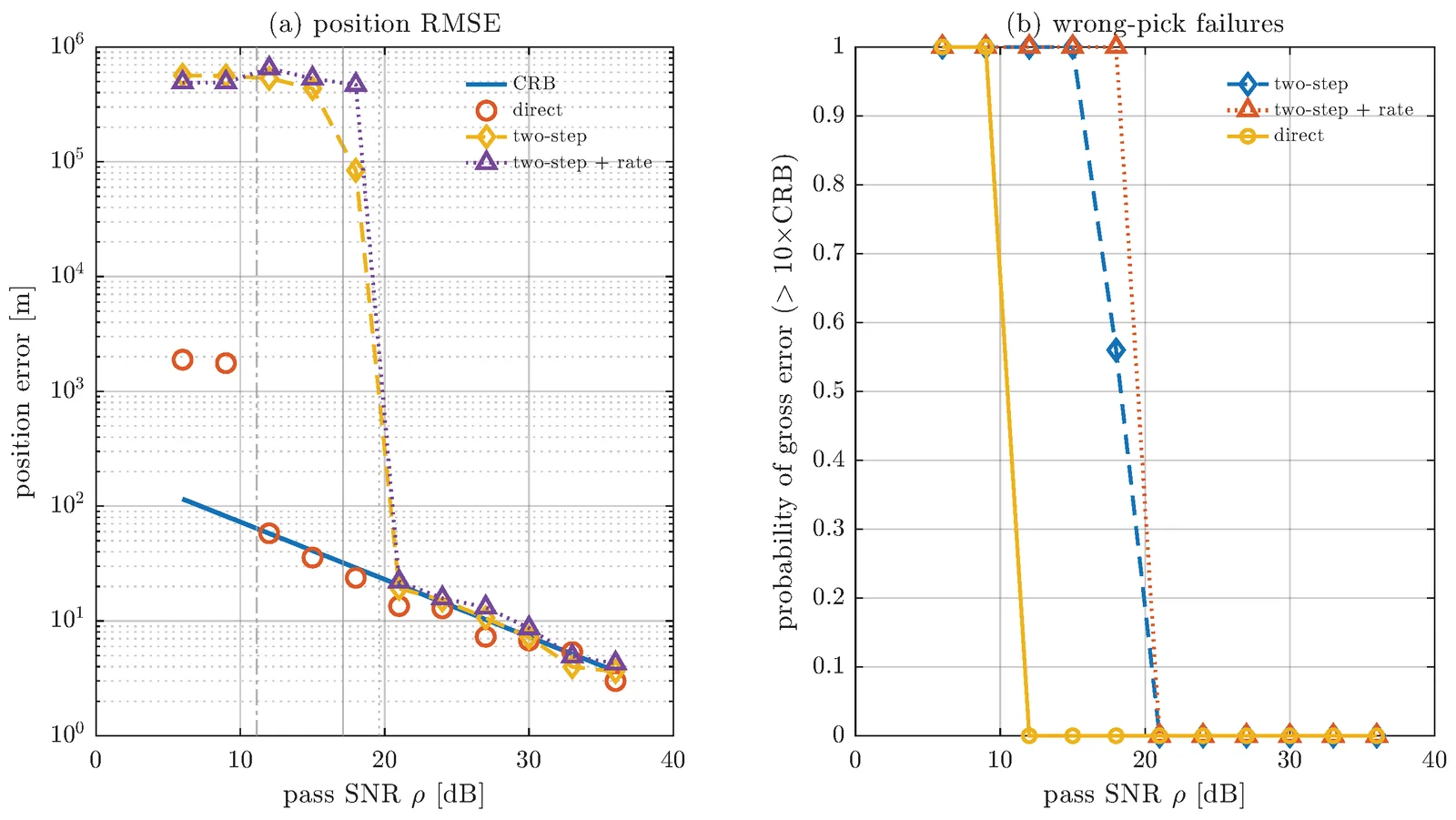
Model-based threshold comparison for a single emitter. Both estimators are assigned acquisition (wrong-peak) failures from the same independent-cell model: the direct estimator by (20), one decision over the two-line-element state box; the two-step estimators by (18), one decision per station and sub-window. (**a**) Root-mean-square position error over all trials versus pass SNR (two-step failures capped at $10^{6}$ m): CRB (line); direct estimator (circles); two-step with delay and Doppler (dashed, diamonds) and the Doppler rate additionally extracted (dotted, triangles). Vertical lines: model thresholds, ≈11 dB (direct, dash-dotted), ≈17 and ≈20 dB (two-step, Equation (19)). (**b**) Probability of a gross error (more than ten times the bound). Above its threshold each estimator coincides with the CRB. The failures are imposed by the model; the underlying surrogate likelihood is unimodal.

For the present parameters (B=2 MHz, T=60 s, K=4, and a 2km×2kHz coarse-ephemeris box) this gives $N_{\mathrm{cell}}\approx 4\times 10^{5}$ and $\rho^{★}\approx 17$ dB. This onset of order-unity wrong-pick probability is denoted $\rho_{\mathrm{onset}}\approx 17$ dB; a practical low-error operating point ($P_{\mathrm{wrong}}\leq 10^{-2}$) lies about 1.5 dB higher, $\rho_{1\%}\approx 18.5$ dB. A two-step estimator can, of course, also extract a *third* observable per sub-window—the Doppler rate—to use more of the information that the single-step estimator exploits. This does not remove the threshold; it raises it. The search becomes three-dimensional, adding a Doppler-rate factor $\Delta \dot{f}\;T_{\mathrm{sub}}^{2}/2$ to (17), so $N_{\mathrm{cell}}$ grows by several orders of magnitude (to $∼\;9\times 10^{9}$ for the same parameters with a Doppler-rate search span $\Delta \dot{f}=200$ Hz/s, which covers the LEO Doppler-rate range at 2.2 GHz) and, by (19), the threshold rises to $\rho^{★}\approx 20$ dB. The extra observable improves the above-threshold accuracy (the fit uses more information) but worsens the detection SNR required to obtain it—a direct expression of the short-window/long-window tension noted above, now sharpened because resolving frequency curvature demands still longer windows. Figure 11 shows both two-step variants. (No claim is made that the three extracted observables retain all of the information in the raw likelihood; the higher-order baseline is the more complete of the two two-step schemes tested, not an information-equivalent one.) Below $\rho^{★}$, under a uniform-wrong-pick model, a wrong selection can inject range errors of order cΔτ, which map into large orbital-state position errors depending on the geometry; above it the picks are reliable and the two-step estimate attains the CRB.

The direct estimator is subject to the same mechanism, but it makes a single peak decision on the concentrated statistic (5) of the whole arc and all stations over the $N_{\mathrm{cell}}^{\mathrm{dir}}\approx 10^{14}$ state cells of the two-line-element box. A noise cell of that statistic, an incoherent sum over *M* stations, is Γ(M,1)-distributed. In the same fixed-signal-cell approximation it exceeds the signal level Mρ with probability

(20) $$P_{1}^{\mathrm{dir}}=e^{-M\rho }\sum_{k=0}^{M-1}\frac{{\left(M\rho \right)}^{k}}{k!},\;P_{\mathrm{wrong}}^{\mathrm{dir}}=1-{\left(1-P_{1}^{\mathrm{dir}}\right)}^{N_{\mathrm{cell}}^{\mathrm{dir}}-1}.$$

For M=3 this crosses order unity at $\rho_{\mathrm{dir}}^{★}\approx 11$ dB, and $P_{\mathrm{wrong}}^{\mathrm{dir}}\leq 10^{-2}$ at 11.6 dB: the far larger number of cells is more than offset by using the energy of the whole arc and all the stations in one decision, whereas the two-step estimator decides per station on a quarter of the energy. The direct threshold of (20) assumes an exhaustive search; a hierarchical acquisition reintroduces intermediate decisions at its coarse stage, so $\rho_{\mathrm{dir}}^{★}$ is a best case, and the practical advantage lies between zero and the model value.

Both estimators estimate $\delta t_{\mathrm{tx}}$ jointly with the state. In the Monte Carlo experiment of Figure 11 both are assigned acquisition failures from these models: the two-step per-sub-window extraction fails with probability (18) (a wrong pick being uniform in the search box) before the orbit fit, and the direct estimator fails with probability (20) (a wrong pick being uniform in the state box, which leaves an error of the order of kilometers); otherwise each proceeds to its orbit fit. The experiment reproduces the model thresholds and the convergence to the CRB above them: the probability of a gross error (more than ten times the bound) is one up to ρ=15 dB, 0.56 at 18 dB and zero from 21 dB for the delay/Doppler baseline and one up to 18 dB and zero from 21 dB for the Doppler-rate variant. For the direct estimator every trial at 6 and 9 dB is a wrong pick, with errors of 1–2 km, and there are no gross errors from 12 dB. Above threshold the root-mean-square error follows the bound (within the Monte Carlo scatter of 15 trials per point). Because a two-step wrong pick produces errors of hundreds of kilometers, the two-step RMSE below threshold is dominated by the failures (each capped at $10^{6}$ m, the order of the orbital error that the search-box extent maps to) and is reported together with the gross-error probability, which shows the mechanism. Under this common model the direct estimator’s threshold lies about 6 dB below that of the delay/Doppler two-step baseline and about 8.5 dB below that of the Doppler-rate variant. The advantage is thus an extension of usable operation into the weak-signal regime, not a lower asymptote: the two approaches coincide where observables are reliably extractable. This advantage is predicted by the failure model, not demonstrated by a simulation of the raw-sample likelihood and its acquisition, which is left to future work. This comparison is against a two-step architecture that commits to a hard peak decision per sub-window; a more elaborate scheme retaining soft likelihoods or multiple candidate peaks could lower its threshold, so the precise claim is that direct processing avoids the threshold associated with hard intermediate peak decisions and replaces it with the lower threshold of a single decision on the full-arc statistic. A structural point underlies this: two-step processing is caught between using short sub-windows, needed to track the rapidly varying Doppler, and long ones, needed for reliable detection—a tension the single-step estimator avoids at the level of its final decision, although a practical hierarchical acquisition reintroduces sub-window processing at its coarse stage.

### 7.11. Summary of Accuracy

Table 4 collects the principal quantitative results for the scenario of this section.

## 8. Conclusions

This paper formulated the single-step direct determination of a non-cooperative LEO satellite’s orbital state from multi-station RF signals, inverting the usual direct-geolocation roles so that the satellite is the unknown emitter. Because the trajectory is a deterministic function of the epoch state, the estimator searches only over valid orbits, with the orbital-dynamics manifold intrinsic to the parameterization. The orbital-state Cramér–Rao bound was derived and shown to factor into signal, geometry, and dynamics terms, the last being the two-body state-transition matrix for which a closed-form position block was given via implicit differentiation of the universal Kepler equation. The delay model evaluates the satellite position at the emission epoch (light-time correction), which leaves the bound essentially unchanged but removes a bias of more than a hundred meters. The unknown emission epoch of a non-cooperative emitter enters as a common time-offset nuisance so that, with synchronized stations, the delays carry time-difference information; this roughly doubles the three-station bound on the near-collinear baseline, whereas four stations recover the known-epoch accuracy. Simulations validate the analytical derivation under the stated model assumptions, demonstrate post-acquisition efficiency against the deterministic CRB under a Gaussian delay/Doppler surrogate of the known-waveform model, and, under a common model of acquisition failures, place the direct estimator’s threshold about 6 dB below that of a two-step baseline that commits to hard per-window peak decisions; this advantage is model-based, an upper limit for a practical hierarchically acquired direct estimator, and remains to be confirmed by a raw-I/Q simulation; more elaborate two-step schemes with soft or multiple-hypothesis processing may narrow the gap. The stochastic unknown-waveform FIM retains the same geometry/dynamics structure with an information factor γ/(1+γ) in the per-sample array SNR, which confines the unknown-waveform estimator to high per-sample SNR; at low-pass SNRs where the single-step advantage is predicted, the waveform must be known or structured. The station-count conclusions were established on a nondimensionalized information matrix for the regional layouts and passes tested, and well-separated non-collinear stations were found to be worth as much as an added station. The limitations include the computational cost of the six-dimensional search (mitigated by coarse initialization and the smoothness of the orbit), the assumption of Keplerian dynamics in the closed-form bound (relaxed numerically via the variational equations), and a single-emitter model. The most consequential extension is to the multiple-emitter case—for example, a satellite downlink coincident in frequency and time with a co-channel terrestrial interferer, a realistic non-cooperative scenario—where the single-step approach may provide a further signal-to-noise-independent advantage by avoiding the measurement association that two-step processing requires; this hypothesis remains to be quantified in future multi-emitter analysis. Future work will also address maneuvering (non-Keplerian) satellites—linking to thrust estimation for non-cooperative targets—and experimental validation with synchronized multi-station raw-I/Q capture. Public LEO I/Q datasets exist, but the coherent multi-station recordings that are required for experimental validation are not available in the present study and define a concrete next step. 

## Figures and Tables

**Table 1:** Principal notation. Vectors are in the ECI frame of Section 2 unless stated otherwise.

Symbol	Meaning
$t_{0}$; $x={\left[r_{0}^{⊤},v_{0}^{⊤}\right]}^{⊤}$	epoch; orbital state at $t_{0}$ (satellite position $r_{0}$ and velocity $v_{0}$)
ϕ(x,t)	orbit propagator: satellite position at time *t* generated by the epoch state x
$p_{s}\left(t\right)$, $v_{s}\left(t\right)$	satellite position and velocity at time *t*
$r_{0}$, $v_{0}$, $v_{r0}$	$∥r_{0}∥$, $∥v_{0}∥$, and radial velocity $r_{0}^{⊤}v_{0}/r_{0}$ at epoch (Section 5)
μ, $\omega_{⊕}$	Earth’s gravitational parameter; Earth rotation rate
$q_{m}^{\mathrm{ECEF}}$, $q_{m}\left(t\right)$, ${\dot{q}}_{m}\left(t\right)$	position of station *m* in ECEF; its ECI position and velocity
$R_{⊕}\left(t\right)$	rotation from ECEF to ECI about the polar axis by $\omega_{⊕}\left(t-t_{0}\right)$
*M*	number of ground stations
$\tau_{m}\left(t\right)$, $t_{e}$	propagation delay to station *m* at reception time *t*; emission epoch $t_{e}=t-\tau_{m}\left(t\right)$
$d_{m}$, ${\hat{u}}_{m}$	line-of-sight vector $\phi \left(x,t_{e}\right)-q_{m}\left(t\right)$ and its unit vector
s(t), $f_{c}$, *B*	complex envelope; carrier frequency; rms bandwidth
$b_{m}$, $N_{0}$	complex gain of station *m*; noise power spectral density
$\delta t_{\mathrm{tx}}$	unknown emission-time offset (common to all stations)
$\delta f_{m}$, ${\dot{f}}_{\mathrm{tx}}$	identifiable per-station frequency offset; common transmitter drift
J, C	Fisher information matrix; Cramér–Rao bound $J^{-1}$
$\Phi (t,t_{0})$, $\Phi_{\;r}\left(t,t_{0}\right)$	state-transition matrix $\partial (p_{s},v_{s})/\partial x$ and its position block $\partial p_{s}/\partial x$
χ, α, C(z), S(z)	universal anomaly; reciprocal semi-major axis; Stumpff functions
ρ, $\rho_{s}$	pass SNR per station; per-sample SNR (ρ/N per raw sample, $\rho /N_{s}$ in the surrogate (16))
*N*, $N_{s}$, *T*	raw samples per station; delay/Doppler samples of the numerical surrogate; arc duration
*K*, $T_{\mathrm{sub}}$	number of sub-windows of the two-step baseline; sub-window duration T/K
a(x), R(x)	manifold vector and spatial covariance of the unknown-waveform model (Section 6)

**Table 2:** Layered modeling assumptions.

Layer	Assumptions
Core idealized model	free-space propagation; white Gaussian noise; two-body (Keplerian) orbit; known station positions
Known-waveform model	transmitted waveform known (deterministic)
Unknown-waveform model	zero-mean complex-Gaussian signal; geometric information via delay and Doppler; no carrier phase
Nuisance model	per-station carrier offset $\delta f_{m}$; common transmitter drift ${\dot{f}}_{\mathrm{tx}}$; common emission-time offset $\delta t_{\mathrm{tx}}$ (stations synchronized; station clock offsets and drift discussed in Section 4)
Station geometry	known station coordinates; Earth rotation included ($q_{m}\left(t\right)$, ${\dot{q}}_{m}$ in ECI); timing/frequency errors as nuisances
Numerical scenario	M=3 stations; 543 km, $65^{\circ }$ orbit; $f_{c}=2.2$ GHz; B=2 MHz rms; 60 s arc; equal per-station SNR; Doppler window rms duration $T_{d}=1$ s

**Table 3:** Signal model, nuisance assumptions and bound used in each numerical experiment. “Deterministic” denotes the delay/Doppler surrogate of (9) defined in Section 7.

Experiment	Waveform/Gains	Nuisances	Bound
STM validation (Figure 5)	—	—	analytic vs. finite difference
CRB vs. SNR, efficiency (Figure 6)	known; unknown constant gains	$\delta t_{\mathrm{tx}}$ (frequencies known)	deterministic (9), gains concentrated
Arc length (Figure 7)	known; unknown constant gains	$\delta t_{\mathrm{tx}}$	deterministic
Frequency nuisances (Figure 1 and Figure 2)	known; unknown constant gains	$\delta t_{\mathrm{tx}}$, $\delta f_{m}$, ${\dot{f}}_{\mathrm{tx}}$ (Schur)	deterministic, Schur-complemented
Path loss (Figure 3)	known; range-weighted gains	$\delta t_{\mathrm{tx}}$/+ frequencies	deterministic
Stations, observability (Figure 8, Figure 9 and Figure 10)	known	$\delta t_{\mathrm{tx}}$	deterministic; scaled FIM spectrum
Light time (Section 7)	known	$\delta t_{\mathrm{tx}}$	deterministic, retarded vs. simultaneous
Two-step comparison (Figure 11)	known	$\delta t_{\mathrm{tx}}$	deterministic
Unknown waveform (Section 6)	Gaussian, unknown	none	stochastic (15), analytic only

**Table 4:** Summary of the orbital-state accuracy obtained in this section (pass SNR ρ=15 dB per station, 60 s arc, and synchronized stations with the emission-time offset estimated unless stated otherwise). Position values are $\sqrt{\mathrm{tr}C_{rr}}$.

Case	Result
Baseline network, M=3, frequencies known: position/velocity CRB	41 m/0.15 m/s
Direct estimator after acquisition, Monte Carlo RMSE, same case	45 m
Same, ρ=33 dB: CRB/Monte Carlo RMSE	5.2 m/4.7 m
Frequency offsets and drift also estimated, M=3/4/6	78/17/12 m
Baseline network plus a fourth station, frequencies known	7.0 m
Layouts, M=3: 300 km triangle/600 km triangle/300 km square	13/5/7 m
Arc length (fixed energy): 10 s/45 s/90 s	80/42/38 m
Bias of the simultaneous-time (no light-time) model	276 m, 1.04 m/s
Acquisition threshold (model): direct/two-step/two-step with Doppler rate	11/17/20 dB

## Data Availability

No observational data were collected in this study; all the results are simulations whose inputs are specified in Section 7 and Table 2 and Table 3. The MATLAB (R2025b) script that implements the state-transition matrix, Fisher information, and CRB with the light-time model and direct estimator and two-step baseline and produces every figure and number quoted in Section 7 with a fixed random seed is available from the author upon reasonable request.
